# Gallbladder microbiota in healthy dogs and dogs with mucocele formation

**DOI:** 10.1371/journal.pone.0281432

**Published:** 2023-02-10

**Authors:** Jody L. Gookin, Ashley N. Hartley, Kathleen M. Aicher, Kyle G. Mathews, Rachel Cullen, John M. Cullen, Benjamin J. Callahan, Devorah M. Stowe, Gabriela S. Seiler, Megan E. Jacob, Jason W. Arnold, M. Andrea Azcarate-Peril, Stephen H. Stauffer

**Affiliations:** 1 Department of Clinical Sciences, College of Veterinary Medicine and Comparative Medicine Institute, North Carolina State University, Raleigh, North Carolina, United States of America; 2 Department of Population Health and Pathobiology, College of Veterinary Medicine, North Carolina State University, Raleigh, North Carolina, United States of America; 3 Department of Molecular Biomedical Sciences, College of Veterinary Medicine, North Carolina State University, Raleigh, North Carolina, United States of America; 4 Department of Medicine, Division of Gastroenterology and Hepatology, and UNC Microbiome Core, Center for Gastrointestinal Biology and Disease, School of Medicine, University of North Carolina, Chapel Hill, North Carolina, United States of America; Universidade Lisboa, Instituto superior Técnico, PORTUGAL

## Abstract

To date studies have not investigated the culture-independent microbiome of bile from dogs, a species where aseptic collection of bile under ultrasound guidance is somewhat routine. Despite frequent collection of bile for culture-based diagnosis of bacterial cholecystitis, it is unknown whether bile from healthy dogs harbors uncultivable bacteria or a core microbiota. The answer to this question is critical to understanding the pathogenesis of biliary infection and as a baseline to exploration of other biliary diseases in dogs where uncultivable bacteria could play a pathogenic role. A pressing example of such a disease would be gallbladder mucocele formation in dogs. This prevalent and deadly condition is characterized by excessive secretion of abnormal mucus by the gallbladder epithelium that can eventually lead to rupture of the gallbladder or obstruction of bile flow. The cause of mucocele formation is unknown as is whether uncultivable, and therefore unrecognized, bacteria play any systematic role in pathogenesis. In this study we applied next-generation 16S rRNA gene sequencing to identify the culture-negative bacterial community of gallbladder bile from healthy dogs and gallbladder mucus from dogs with mucocele formation. Integral to our study was the use of 2 separate DNA isolations on each sample using different extraction methods and sequencing of negative control samples enabling recognition and curation of contaminating sequences. Microbiota findings were validated by simultaneous culture-based identification, cytological examination of bile, and fluorescence in-situ hybridization (FISH) performed on gallbladder mucosa. Using culture-dependent, cytological, FISH, and 16S rRNA sequencing approaches, results of our study do not support existence of a core microbiome in the bile of healthy dogs or gallbladder mucus from dogs with mucocele formation. Our findings further document how contaminating sequences can significantly contribute to the results of sequencing analysis when performed on samples with low bacterial biomass.

## Introduction

Bile is synthesized by the liver and stored in the gallbladder. In response to ingestion of a meal, the gallbladder contracts and empties bile into the small intestine resulting in the delivery of bile salts that aid in the assimilation of dietary fat. Despite an intense interest in the influence of intestinal microbiota in health and disease, it is surprising that there are few studies investigating the culture-independent microbiome of bile [1–11], none of which has included dogs. In healthy dogs, bile is generally regarded as sterile [12]. What remains unknown is whether bile from healthy dogs harbors uncultivable bacteria or a core microbiota. The answer to this question is critical to understanding the pathogenesis of biliary infection, which is one of the most common and treatable causes of hepatobiliary disease in dogs [13–21].

Recently, a unique gallbladder disease in dogs has gained broad recognition [22–29]. The disorder, referred to as gallbladder mucocele formation, is characterized by excessive secretion of abnormal mucus by the gallbladder epithelium [30]. This mucus can eventually lead to rupture of the gallbladder or obstruction of bile flow [30]. The cause of mucocele formation is unknown. In dogs undergoing surgery for removal of a gallbladder mucocele, routine aerobic and anaerobic culture of gallbladder content identifies the presence of common biliary pathogens such as *E*. *coli* and *Enterococcus* spp. in anywhere from 2.7 to 67% (median, 13%) of cases [22, 23, 25, 27, 28, 31–34]. What is unknown is whether uncultivable, and therefore unrecognized bacteria, play any systematic role in pathogenesis of gallbladder mucocele formation.

Studies examining the bile microbiome in humans are far from any consensus. Conclusions range from there being no bile microbiome [7] to the existence of a bile microbiome that rivals the number and diversity of taxa found in the intestinal tract [4]. Limitations with studies in humans include the inability to collect bile from otherwise healthy individuals or use of collection methods that are not aseptic such as retrograde catheterization of the common bile duct from within the duodenum. In addition, many studies do not describe controls for the impact of laboratory DNA contamination. It is now well recognized that reagent and laboratory DNA contamination can significantly influence the results of microbiota studies, particularly when samples have a low microbial biomass [35, 36]. Under these circumstances, contaminating DNA can outcompete low copy number sample DNA, swamping the amplification process, and resulting in spurious sequencing results.

Our objective in this study was to apply next-generation 16S rRNA gene sequencing to identify the culture-negative bacterial community of gallbladder bile from healthy dogs and gallbladder mucus from dogs with mucocele formation. Our specific aims were to determine if a core bile microbiome could be identified in healthy dogs and whether dogs with mucocele formation were systematically colonized by previously unrecognized bacterial taxa. Integral to our study was inclusion in the analysis of (1) two independent DNA isolations on each sample, (2) two different extraction methods, and (3) sequencing of negative controls from different stages in the process, enabling recognition and curation of contaminating sequences. Microbiota findings were validated by simultaneous culture-based identification, cytological examination of bile, and fluorescence in-situ hybridization performed on gallbladder mucosa.

## Methods

### Sample collection

#### Control dogs

Apparently healthy purpose-bred research dogs were scheduled to undergo euthanasia for purposes of colony depopulation unrelated to this study at which time tissues became available for collection. Dogs relinquished to animal control and undergoing euthanasia for reasons related to aggressiveness toward humans or other dogs were sampled when available. Immediately after intravenous injection of a lethal dose of pentobarbital and auscultatory confirmation of cardiac arrest, the abdomen was opened via right flank abdominal incision without sterile preparation. With the incision held open, the gallbladder was visualized in the cranial abdomen and aspirated using a 12 cc syringe with 18-gauge needle without contacting the body wall or other internal organs. Bile was transferred within 10 minutes to a sterile microcentrifuge tube and frozen in liquid nitrogen prior to storage at -80°C. The gallbladder was subsequently removed from the abdominal cavity and a 1 x 1 inch square sheet of mucosa was excised and placed in 10% neutral-buffered formalin.

#### Dogs with gallbladder mucocele formation

Dogs with gallbladder mucocele formation were client-owned patients of either of two participating specialty referral hospitals where mucocele formation was diagnosed by means of ultrasonographic criteria [27] and owners elected surgical removal of the gallbladder. Gallbladders were obtained intraoperatively immediately after cholecystectomy. While still in the operating room, the gallbladder was transected using sterile instruments. All gallbladders were distended with intraluminal solidified mucus as is pathognomonic of mucocele formation. A sample of the lumen mucus content was placed into a sterile microcentrifuge tube and frozen at -80°C. A 1 x 1 inch square sheet of mucosa was excised from the gallbladder and placed in 10% neutral-buffered formalin.

#### Ethics statement

This study was carried out in strict accordance with the recommendations in the Guide for the Care and Use of Laboratory Animals of the National Institutes of Health. The protocol was approved by the Institutional Animal Care and Use Committee of North Carolina State University. (Protocol Number: 14–049).

### Cytological examination

Bile samples collected from control dogs underwent cytological examination by an American College of Veterinary Pathology certified study investigator (D.S.). Each cytological specimen was prepared at the time of bile collection by smearing a drop of bile onto each of 2 individual glass microscope slides. Slides were air-dried, fixed, and stained with hematoxylin and eosin. Samples of gallbladder mucus from dogs with mucocele formation were not examined cytologically.

### Bacterial culture

For aerobic and anaerobic culture, samples of bile or mucus were directly plated on Columbia agar with 5% sheep blood, MacConkey agar (Remel; Lenexa, KS 66215) and pre-reduced Brucella blood agar plates (BBE/LKV agar; Anaerobe systems, Morgan Hill, CA 95037). Additionally, samples were enriched in chopped meat broth. All plates and enrichments were incubated at 36°C in 5% CO_2_ (blood agar and MacConkey agar) or in an anaerobic chamber (BBE/LKV and enrichment). All cultures were examined for growth daily for up to 5 days. Bacterial isolates were isolated and identified by laboratory standard operating procedures, including MALDI-TOF. At least one subculture of enrichment was performed during the growth period.

### Medical records review

Medical records of client-owned dogs were reviewed for collection of age, breed, sex, neuter-status, and history of antibiotic administration within the prior 2 week period. Ultrasonographic images of the gallbladder were reviewed by an American College of Veterinary Radiology certified study investigator (G.S.) and histology findings from submitted surgical samples of the gallbladder were reviewed to confirm the final diagnosis of gallbladder mucocele formation.

### Fluorescence in-situ hybridization

Formalin-fixed samples of gallbladder mucosa from control dogs and dogs with mucocele formation were embedded in paraffin, sectioned at thickness of 4 μm, mounted on poly-L-lysine coated slides, and fluorescence *in situ* hybridization (FISH) was performed as previously described [37, 38]. Probes used for hybridization included a universal eubacterial probe, Eub338 (5’-GCTGCCTCCCGTAGGAGT-6-FAM-3’, where 6-FAM is 6-carboxyfluorescein) and a negative-control non-Eub probe (5’-Cy3-CGACGGAGGGCQTCCTCA-3’). The probes were reconstituted with sterile water and diluted with hybridization buffer to final working concentrations of 5 ng/μl. Formalin-fixed and paraffin-embedded intestinal tissue from a piglet diagnosed with necrotizing enterocolitis was included in each hybridization experiment as a positive control.

### Bacterial 16S rRNA gene amplification and sequencing

#### DNA extraction

To aid in distinguishing genuine signals from reagent-derived signals, we performed two separate DNA isolations on each sample using reagents from kits produced by different suppliers [36]. All DNA extraction steps were performed in a laminar flow tissue culture hood with exception to steps involving use of phenol, which were performed in a chemical safety hood. Samples were thawed, and either aliquoted (500 μl for bile samples) or swabbed (Zymobiomics)(for mucus samples) into 2 separate sterile 2 ml flat screw-cap cryotubes containing mixed diameter glass beads (VWR Micro Centrifuge Tube Cat# 16466–058 and disruptor beads Cat# 30623–118 and -120) and centrifuged for 10 minutes at 20,000×g. Any supernatant was discarded and the pellet was resuspended in 500 μl lysis buffer (Qiagen, ATL buffer). Samples underwent bead-beating on a Vortex Genie for 45 minutes at room temperature followed by addition of 20 μl of proteinase K (Qiagen) and further incubation at 55°C for 8 hours. After incubation all samples underwent an additional bead beating for 5 minutes.

One tube for each sample underwent further extraction using a “no-phenol” (NP) method while the other tube was extracted using a “phenol” (P) method. No-phenol samples were centrifuged for 1 minute at 15,000×g and the supernatant transferred to ½ Inhibix tablet (Qiagen) and vortexed to fully disperse the tablet. The sample was centrifuged at 15,000×g for 2 minutes. The resulting supernatant (300 μl) was mixed well with 300 μl AL buffer (Qiagen) in a clean tube and incubated at 55°C for 8 hours. After incubation samples were mixed well with 300 μl of 100% ethanol and loaded onto a Zymo-Spin IC silica mini-elute column (Cat#C1004-250) and centrifuged at 8,000×g for 1 minute. Columns were washed with 500 μl of AW1 wash buffer (Qiagen) and centrifuged for 1 minute followed by 500 μl of AW2 wash buffer (Qiagen) and centrifugation for 5 minutes. Columns were dried for 10 minutes and DNA was eluted by incubation with 25 μl of PCR-grade water for 5 minutes followed by centrifugation for 2 minutes at 8,000×g.

Duplicate samples extracted using the phenol method were treated with 500 μl of phenol-chloroform and vortexed for 5 minutes. Following centrifugation 10,000×g for 2 minutes the aqueous phase was transferred to a new tube to which 500 μl of chloroform was added followed by vortexing and centrifugation. The aqueous phase was similarly washed again in chloroform prior to transfer to a new tube. Washed samples were mixed well with 2× volume Zymobiomics binding buffer, applied to a Zymo silica column, and centrifuged for 1 minute. The column was washed twice with 500 μl of Zymo wash buffer and allowed to dry for 10 minutes. DNA was eluted from the column by incubation with 25 μl of PCR-grade water for 5 minutes followed by centrifugation for 2 minutes at 8,000×g.

#### Negative extraction controls

For each extraction method (no phenol versus phenol), 6 extraction control tubes treated with only PCR-grade water and lacking the addition of bile or mucus were included in all steps. For each extraction method, 3/6 tubes had a collection swab inserted to account for potential DNA contamination introduced by the swab.

#### 16S rRNA gene amplification and sequencing

Extracted DNA was quantified via PicoGreen analysis and used for bacterial 16S rRNA gene amplicon sequencing as described [39, 40], with specific modification of using qPCR to determine the cycle at which each sample or control began logarithmic amplication. Total DNA (12.5 ng) was amplified using universal primers targeting the V4 region of the bacterial 16S rRNA gene [41]. Overhang adapters were appended to the 5′ end of each primer sequence for compatibility with the Illumina sequencing platform. For each sample, a cycle-optimization qPCR was first performed to determine the cycle at which each began logarithmic amplification. Amplicons from each sample were collected at the optimized cycle to prevent over-cycling of highly concentrated samples while allowing ample time for lower input samples to amplify. Each 16S rRNA DNA amplicon was purified using the AMPure XP reagent (Beckman Coulter, Indianapolis, IN). In the next step each sample was amplified using a limited cycle PCR program, adding Illumina sequencing adapters and dual index barcodes (index 1(i7) and index 2(i5)) (Illumina, San Diego, CA) to the amplicon target. The final libraries were again purified using the AMPure XP reagent (Beckman Coulter), quantified with Quant-iT^™^ PicoGreen^®^ dsDNA Reagent (Molecular Probes, Thermo Fisher Scientific, Waltham, MA), and normalized prior to equimolar pooling. The DNA library pool was then denatured with NaOH, diluted with hybridization buffer and heat denatured before loading on the MiSeq reagent cartridge MiSeq instrument (Illumina). Automated cluster generation and paired–end sequencing with dual reads were performed according to the manufacturer’s instructions.

#### Sequencing data analysis

Sequencing output from the Illumina MiSeq platform were converted to fastq format and demultiplexed using Illumina Bcl2Fastq 2.20.0.422. Paired end reads were classified with Kraken2 [42] and all reads identified as being derived from the *Canis lupus familiaris* reference Cf31, the *Canis lupus* mitochondrial reference, or the Human Hg19 reference were eliminated. The resulting paired-end reads were processed with the QIIME 2 2018–6 [43] wrapper for DADA2 [44] including merging paired ends, quality filtering, error correction, and chimera detection. Amplicon sequencing units from DADA2 were assigned taxonomic identifiers with respect to the Silva 138 database [45]. Results were compared to a second assessment using the Greengenes [46] database with no substantial observed difference. Sequences were aligned using maFFT [47] in QIIME 2, and a phylogenetic tree was built with FastTree [48] in QIIME 2. Amplicon sequencing units that could not be resolved with Green Genes or Silva were submitted to Kraken2 [42] with the standard database supplemented with fungal references. Alpha diversity with respect to: Faith index and Evenness index; was estimated using QIIME 2 at a rarefaction depth of both 1,000 and 5,000 sequences per subsample. Beta diversity estimates were calculated within QIIME 2 using weighted Unifrac distances [49] and Bray Curtis distance [50] between samples at a subsampling depths of 1,000 and 5,000 separately. Results were summarized and visualized through principal coordinate analysis as implemented in QIIME 2.

#### Curation of sequence reads

Taxa amplified from any negative control samples under any extraction conditions were designated as DNA contaminants. Contaminating taxa were excluded from patient sample analysis when present at a read count < 10 or ≤ the maximum read count observed in any negative control samples. As described by others working with sequence data from bile samples [1, 6], taxa not identified in negative extraction controls but with low read counts (≤ 10 reads in all patient samples) were not included in sample analysis.

#### Core microbiota

For the purpose of this study, members of a core microbiome were defined as ASV not designated as a DNA contaminant that were amplified using both extraction methods from the same sample, and detected in at least 30% of samples.

## Results

### Contaminating sequence data

**Fig 1:**
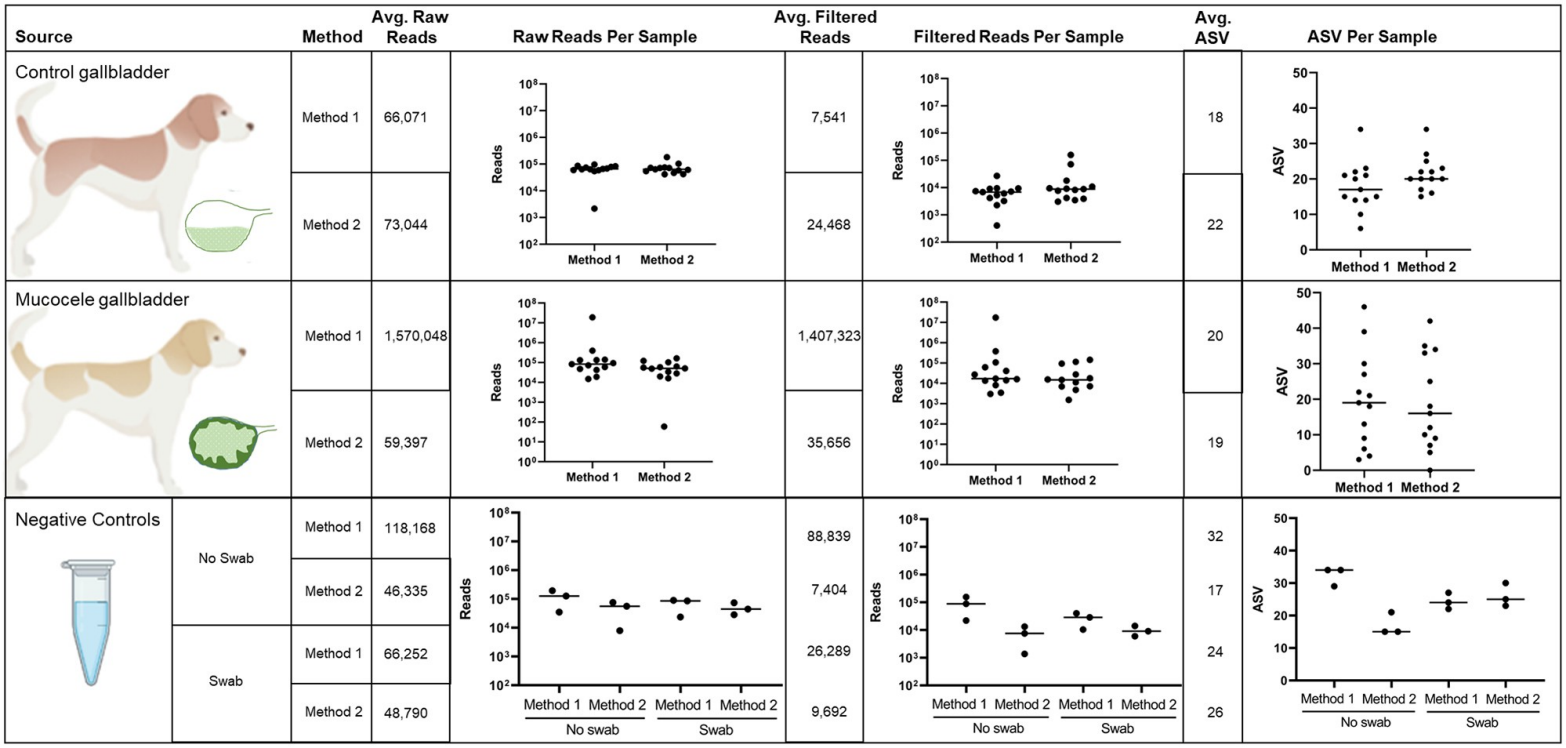
Number of sequencing reads and amplicon sequence variants (ASVs) identified in 13 samples of bile from the gallbladder of healthy control dogs, 13 samples of mucus from the gallbladder of dogs with mucocele formation and 4 negative extraction controls run in triplicate under each of two extraction protocols with or without introduction of a sterile swab. Method 1 DNA extraction performed using phenol. Method 2 DNA extraction performed without use of phenol as described in Methods.

To enable identification and filtering of expected reagent and laboratory DNA contaminants from analysis of gallbladder bile and mucus sample sequencing results, negative extraction control samples were processed through two DNA isolation conditions and subject to 16S rRNA gene amplicon sequencing. Reads aligning against the *Canis lupus familiaris* reference Cf31, the *Canis lupus* mitochondrial reference, and Human Hg19 reference accounted for a median of 44 reads per control sample (range, 0–5,757 reads) and 709 reads per mucocele sample (range, 0–39,105 reads). These reads represented a median of 0.113% of total raw reads (range, 0–7%) in control samples and 2.3% of total raw reads (range, 0.0–30.5%) in mucocele samples. The resulting average and per sample raw and post-processing read counts and total ASVs identified under each extraction condition in experimental and negative control samples are shown in Fig 1.

**Fig 2:**
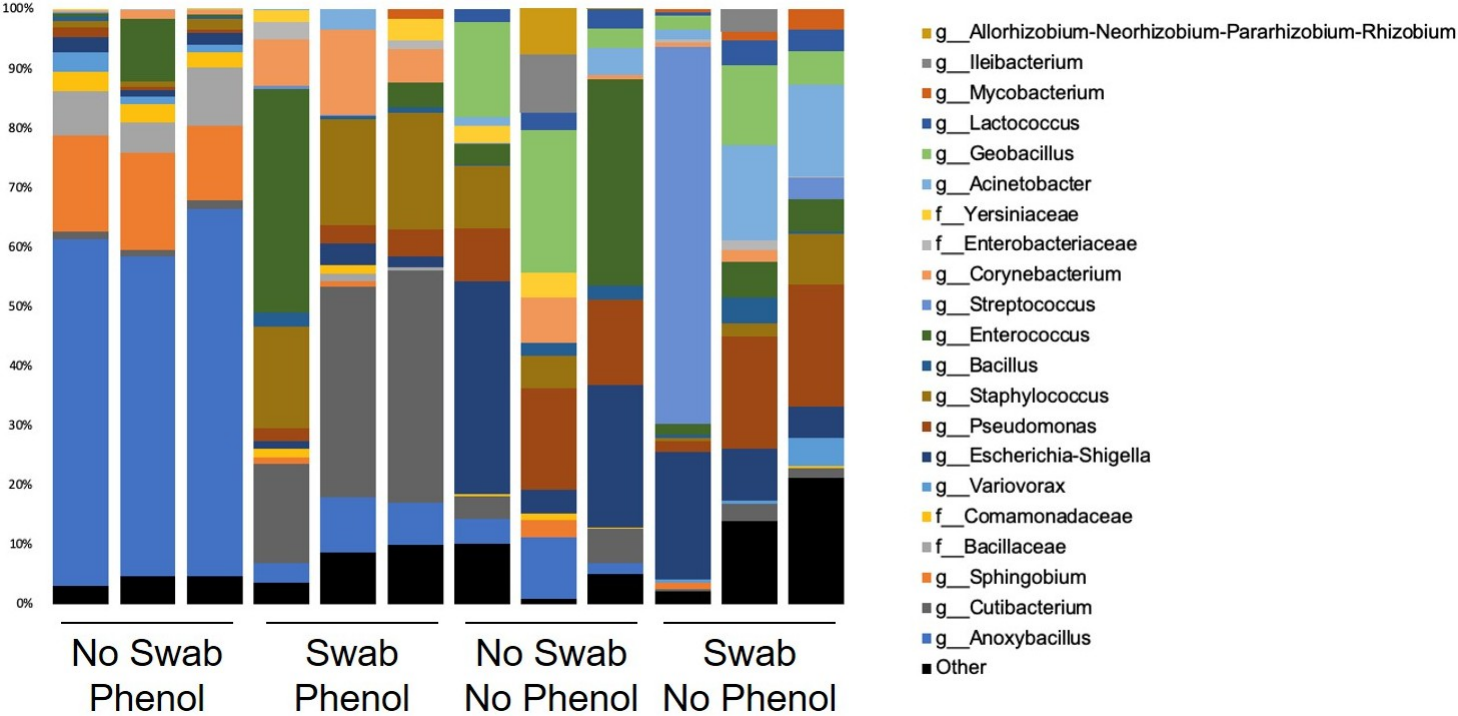
Percent abundance of taxa for which 16S rRNA sequences were amplified from negative extraction control samples under each of 4 DNA isolation conditions.

A total of 108 different ASVs were amplified from ≥ 1 negative extraction control sample and therefore designated as laboratory or reagent DNA contaminants (Fig 2 and S1 Table). Seventy three (67%) of the ASVs identified in negative extraction controls were also amplified from one or more study samples.

### Control dogs

Bile samples were collected from 13 apparently healthy control dogs. Ten dogs underwent euthanasia by animal control and 3 were purpose-bred research dogs euthanized for reasons unrelated to the study. Represented breeds were Staffordshire terrier mix (7 dogs), Hound (3 dogs), Beagle, Border Collie mix, and German Shepherd (1 dog each). Median age was 2.5 years (range, 1 to 4 years).

#### Microbiota composition of bile

After filtration of contaminating sequences and taxa with read counts ≤10 in all samples, control bile samples yielded a median of 3,686 reads (range, 203 to 157,730 reads) using the non-phenol extraction method and a median of 1,439 reads (range, 3 to 14,294 reads) using the phenol extraction method. A total of 94 unique ASVs were identified among all control bile samples. Sixty-seven (71%) of these ASVs were not amplified from any negative extraction control samples (Table 1).

**Table 1:** Sixty-seven amplicon sequence variants amplified from bile collected from 13 apparently healthy adult dogs and not observed in extraction control samples not containing bile.

Amplicon Sequence Variants	Dogs (n = 13)		Max % Abundance
	No.	%	
d__Bacteria;p__Proteobacteria;c__Gammaproteobacteria;o__Xanthomonadales;f__Xanthomonadaceae;g__Stenotrophomonas	4	31	15
d__Bacteria;p__Firmicutes;c__Bacilli;o__Bacillales;f__Bacillaceae;g__Bacillus;s__Bacillus_halodurans	3	23	19
d__Bacteria;p__Proteobacteria;c__Alphaproteobacteria;o__Sphingomonadales;f__Sphingomonadaceae;g__Novosphingobium	3	23	8.8
d__Bacteria;p__Proteobacteria;c__Gammaproteobacteria;o__Burkholderiales;f__Oxalobacteraceae;g__Massilia	3	23	63
d__Bacteria;p__Actinobacteriota;c__Actinobacteria;o__Bifidobacteriales;f__Bifidobacteriaceae;g__Gardnerella	2	15	26
d__Bacteria;p__Actinobacteriota;c__Actinobacteria;o__Corynebacteriales;f__Corynebacteriaceae;g__Turicella;s__uncultured_bacterium	2	15	2.0
d__Bacteria;p__Bacteroidota;c__Bacteroidia;o__Bacteroidales;f__Bacteroidaceae;g__Bacteroides	2	15	8.0
d__Bacteria;p__Bacteroidota;c__Bacteroidia;o__Bacteroidales;f__Muribaculaceae;g__Muribaculaceae;s__uncultured_bacterium	2	15	2.5
d__Bacteria;p__Bacteroidota;c__Bacteroidia;o__Sphingobacteriales;f__Sphingobacteriaceae;g__Sphingobacterium	2	15	15
d__Bacteria;p__Firmicutes;c__Clostridia;o__Peptostreptococcales-Tissierellales;f__Peptostreptococcales-Tissierellales;g__Peptoniphilus	2	15	33
d__Bacteria;p__Firmicutes;c__Negativicutes;o__Veillonellales-Selenomonadales;f__Veillonellaceae;g__Veillonella	2	15	34
d__Bacteria;p__Proteobacteria;c__Gammaproteobacteria;o__Burkholderiales;f__Alcaligenaceae;g__Achromobacter	2	15	2.3
d__Bacteria;p__Proteobacteria;c__Gammaproteobacteria;o__Burkholderiales;f__Comamonadaceae;g__Delftia	2	15	10
d__Bacteria;p__Proteobacteria;c__Gammaproteobacteria;o__Burkholderiales;f__Neisseriaceae;g__Neisseria	2	15	27
d__Bacteria;p__Proteobacteria;c__Gammaproteobacteria;o__Pseudomonadales;f__Moraxellaceae;g__Enhydrobacter	2	15	2.3
d__Bacteria;p__Spirochaetota;c__Spirochaetia;o__Spirochaetales;f__Spirochaetaceae;g__uncultured;s__uncultured_spirochete	2	15	100
d__Bacteria;p__Actinobacteriota;c__Actinobacteria;o__Corynebacteriales;f__Corynebacteriaceae;g__Corynebacterium;s__Corynebacterium_kroppenstedtii	1	8	1.5
d__Bacteria;p__Actinobacteriota;c__Actinobacteria;o__Frankiales;f__Sporichthyaceae;g__Sporichthya	1	8	2.3
d__Bacteria;p__Actinobacteriota;c__Actinobacteria;o__Micrococcales;f__Cellulomonadaceae;g__Pseudactinotalea;s__uncultured_bacterium	1	8	0.8
d__Bacteria;p__Actinobacteriota;c__Actinobacteria;o__Micrococcales;f__Dermatophilaceae	1	8	1.5
d__Bacteria;p__Actinobacteriota;c__Actinobacteria;o__Micrococcales;f__Intrasporangiaceae	1	8	2.2
d__Bacteria;p__Actinobacteriota;c__Actinobacteria;o__Micrococcales;f__Intrasporangiaceae;g__Oryzihumus;s__uncultured_bacterium	1	8	26
d__Bacteria;p__Actinobacteriota;c__Actinobacteria;o__Propionibacteriales;f__Nocardioidaceae;g__Nocardioides;s__uncultured_bacterium	1	8	36
d__Bacteria;p__Actinobacteriota;c__Thermoleophilia;o__Solirubrobacterales;f__Solirubrobacteraceae;g__Solirubrobacter	1	8	1.4
d__Bacteria;p__Bacteroidota;c__Bacteroidia;o__Bacteroidales;f__Prevotellaceae;g__Prevotella;s__Prevotella_melaninogenica	1	8	21
d__Bacteria;p__Bacteroidota;c__Bacteroidia;o__Chitinophagales;f__Chitinophagaceae;g__uncultured	1	8	5.0
d__Bacteria;p__Bacteroidota;c__Bacteroidia;o__Cytophagales;f__Hymenobacteraceae;g__Hymenobacter;s__uncultured_bacterium	1	8	2.9
d__Bacteria;p__Bacteroidota;c__Bacteroidia;o__Flavobacteriales;f__Flavobacteriaceae;g__Capnocytophaga;s__Capnocytophaga_ochracea	1	8	0.15
d__Bacteria;p__Bacteroidota;c__Bacteroidia;o__Flavobacteriales;f__Weeksellaceae;g__Chryseobacterium	1	8	14
d__Bacteria;p__Campilobacterota;c__Campylobacteria;o__Campylobacterales;f__Campylobacteraceae;g__Campylobacter;s__Campylobacter_ureolyticus	1	8	0.38
d__Bacteria;p__Cyanobacteria;c__Cyanobacteriia;o__Cyanobacteriales;f__Chroococcidiopsaceae;g__Chroococcidiopsis_SAG_2023;s__uncultured_cyanobacterium	1	8	0.45
d__Bacteria;p__Deinococcota;c__Deinococci;o__Thermales;f__Thermaceae;g__Meiothermus	1	8	42
d__Bacteria;p__Firmicutes;c__Bacilli;o__Bacillales	1	8	2.4
d__Bacteria;p__Firmicutes;c__Bacilli;o__Bacillales;f__Bacillaceae;g__Bacillus;s__Bacillus_coagulans	1	8	25
d__Bacteria;p__Firmicutes;c__Bacilli;o__Bacillales;f__Planococcaceae;g__Lysinibacillus	1	8	2.6
d__Bacteria;p__Firmicutes;c__Bacilli;o__Lactobacillales;f__Carnobacteriaceae;g__Carnobacterium	1	8	10
d__Bacteria;p__Firmicutes;c__Bacilli;o__Lactobacillales;f__Carnobacteriaceae;g__Granulicatella	1	8	67
d__Bacteria;p__Firmicutes;c__Bacilli;o__Lactobacillales;f__Enterococcaceae;g__Enterococcus;s__Enterococcus_cecorum	1	8	0.28
d__Bacteria;p__Firmicutes;c__Bacilli;o__Lactobacillales;f__Lactobacillaceae;g__Lactobacillus;s__Lactobacillus_brevis	1	8	4.9
d__Bacteria;p__Firmicutes;c__Clostridia;o__Clostridia;f__Hungateiclostridiaceae;g__Ruminiclostridium	1	8	1.8
d__Bacteria;p__Firmicutes;c__Clostridia;o__Clostridiales;f__Clostridiaceae;g__Clostridium_sensu_stricto_9;s__uncultured_bacterium	1	8	22
d__Bacteria;p__Firmicutes;c__Clostridia;o__Lachnospirales;f__Lachnospiraceae;g__[Ruminococcus]_gauvreauii_group	1	8	23
d__Bacteria;p__Firmicutes;c__Clostridia;o__Peptostreptococcales-Tissierellales;f__Anaerovoracaceae;g__[Eubacterium]_brachy_group;s__Eubacterium_brachy	1	8	1.0
d__Bacteria;p__Firmicutes;c__Clostridia;o__Peptostreptococcales-Tissierellales;f__Peptostreptococcaceae	1	8	1.6
d__Bacteria;p__Firmicutes;c__Clostridia;o__Peptostreptococcales-Tissierellales;f__Peptostreptococcaceae;g__Romboutsia	1	8	0.91
d__Bacteria;p__Firmicutes;c__Clostridia;o__Peptostreptococcales-Tissierellales;f__Peptostreptococcales-Tissierellales;g__Fenollaria;s__uncultured_bacterium	1	8	0.25
d__Bacteria;p__Firmicutes;c__Limnochordia;o__Limnochordales;f__Limnochordaceae;g__Limnochordaceae	1	8	6.6
d__Bacteria;p__Firmicutes;c__Negativicutes;o__Acidaminococcales;f__Acidaminococcaceae;g__Acidaminococcus;s__uncultured_organism	1	8	15
d__Bacteria;p__Myxococcota;c__Polyangia;o__Haliangiales;f__Haliangiaceae;g__Haliangium	1	8	1.4
d__Bacteria;p__Planctomycetota;c__Phycisphaerae;o__Phycisphaerales;f__Phycisphaeraceae;g__SM1A02;s__uncultured_bacterium	1	8	10
d__Bacteria;p__Planctomycetota;c__Phycisphaerae;o__Tepidisphaerales;f__WD2101_soil_group;g__WD2101_soil_group;s__uncultured_bacterium	1	8	6.5
d__Bacteria;p__Planctomycetota;c__Planctomycetes;o__Isosphaerales;f__Isosphaeraceae;g__uncultured	1	8	1.8
d__Bacteria;p__Proteobacteria;c__Alphaproteobacteria;o__Acetobacterales;f__Acetobacteraceae;g__Acidiphilium	1	8	22
d__Bacteria;p__Proteobacteria;c__Alphaproteobacteria;o__Micavibrionales;f__uncultured;g__uncultured	1	8	11
d__Bacteria;p__Proteobacteria;c__Alphaproteobacteria;o__Rhizobiales;f__Hyphomicrobiaceae;g__Hyphomicrobium	1	8	9.4
d__Bacteria;p__Proteobacteria;c__Alphaproteobacteria;o__Rhodobacterales;f__Rhodobacteraceae;g__Paracoccus	1	8	28
d__Bacteria;p__Proteobacteria;c__Alphaproteobacteria;o__Rickettsiales;f__Mitochondria;g__Mitochondria	1	8	26
d__Bacteria;p__Proteobacteria;c__Alphaproteobacteria;o__Rickettsiales;f__Mitochondria;g__Mitochondria;s__Botryosphaeria_dothidea	1	8	5.0
d__Bacteria;p__Proteobacteria;c__Alphaproteobacteria;o__Sphingomonadales;f__Sphingomonadaceae;g__Qipengyuania;s__uncultured_bacterium	1	8	0.29
d__Bacteria;p__Proteobacteria;c__Gammaproteobacteria;o__Alteromonadales;f__Alteromonadaceae;g__Alishewanella;s__uncultured_bacterium	1	8	1.9
d__Bacteria;p__Proteobacteria;c__Gammaproteobacteria;o__Alteromonadales;f__Psychromonadaceae;g__Psychromonas	1	8	15
d__Bacteria;p__Proteobacteria;c__Gammaproteobacteria;o__Burkholderiales;f__Burkholderiaceae;g__Pandoraea;s__Alcaligenes_sp.	1	8	3.7
d__Bacteria;p__Proteobacteria;c__Gammaproteobacteria;o__Burkholderiales;f__Burkholderiaceae;g__Ralstonia	1	8	0.27
d__Bacteria;p__Proteobacteria;c__Gammaproteobacteria;o__Enterobacterales;f__Yersiniaceae;g__Serratia	1	8	6.9
d__Bacteria;p__Proteobacteria;c__Gammaproteobacteria;o__Pasteurellales;f__Pasteurellaceae;g__Haemophilus	1	8	8.3
d__Bacteria;p__Proteobacteria;c__Gammaproteobacteria;o__Pseudomonadales;f__Moraxellaceae;g__Acinetobacter;s__Acinetobacter_radioresistens	1	8	5.3
d__Bacteria;p__Proteobacteria;c__Gammaproteobacteria;o__Pseudomonadales;f__Moraxellaceae;g__Acinetobacter;s__uncultured_rumen	1	8	3.0

d_domain, p_phylum, c_class, o_order, f_family, g_genus, s_species

The remaining 27 ASVs were also amplified from ≥ 1 negative extraction control samples and were retained in the analysis because their read count in bile exceeded the maximum count observed in any negative extraction controls (S2 Table).

The majority of ASVs in Table 1 (51/67; 76%) were observed in only a single bile sample. The largest number of control bile samples observed to share a taxa was 4/13. The shared taxa was a member of the genus *Stenotrophomonas* (range, 31 to 242 reads). Three taxa were observed in 3/13 control bile samples and included *Bacillus halodurans* (range, 32 to 138 reads) and a member of the genus *Novosphingobium* (range, 14 to 295 reads) and genus *Massilia* (range, 35 to 128 reads).

**Fig 3:**
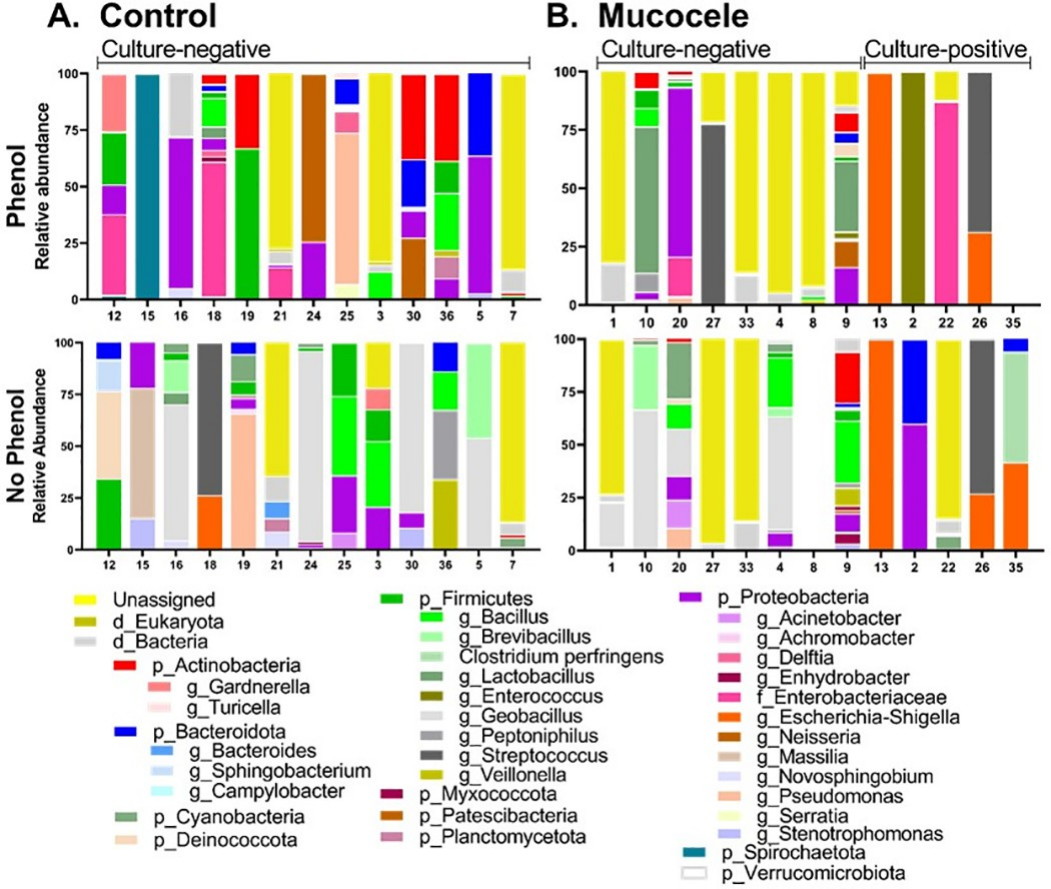
Percent abundance of phyla amplified from the bile of 13 apparently healthy adult dogs (panel A) and gallbladder mucus from 13 dogs with mucocele formation (panel B). Data were filtered for contaminating sequences and taxa with read counts ≤10 in all samples. For each sample, abundance data are shown for sequencing results obtained using each of two different extraction methods (phenol and no-phenol).

The number of unique ASVs per bile sample ranged from 2 to 9 (median, 6) using the non-phenol extraction method and 1 to 17 (median, 5) using the phenol extraction method (Fig 3A and S3 Table). Only 5 of the 94 ASVs were amplified from the same bile sample using both extraction methods. These included a member of the family *Spirochaetaceae* (sample 12; 2 and 36 reads), genus *Novosphingobium* (sample 16; 295 and 14 reads), genus *Bacillus* (sample 3; 1,380 and 664 reads), *Bacillus halodurans* (sample 3; 138 and 65 reads) and genus *Veillonella* (sample 36; 160 and 23 reads).

#### Conventional detection of bacteria

Bacteria were not identified by cytological examination in any control dog bile samples. Yeast organisms were observed in 2 control bile samples (sample 7 and 19). Bacterial growth was not observed in any control dog bile samples under either aerobic or anaerobic culture conditions. Histological examination of the gallbladder was performed in all control dogs, all of which appeared unremarkable and none of which had eubacteria identified by FISH. Combining cytology, conventional culture, and FISH, the presence of bacteria was demonstrated in samples from 0/13 (0%) control dogs.

### Dogs with gallbladder mucocele formation

**Fig 4:**
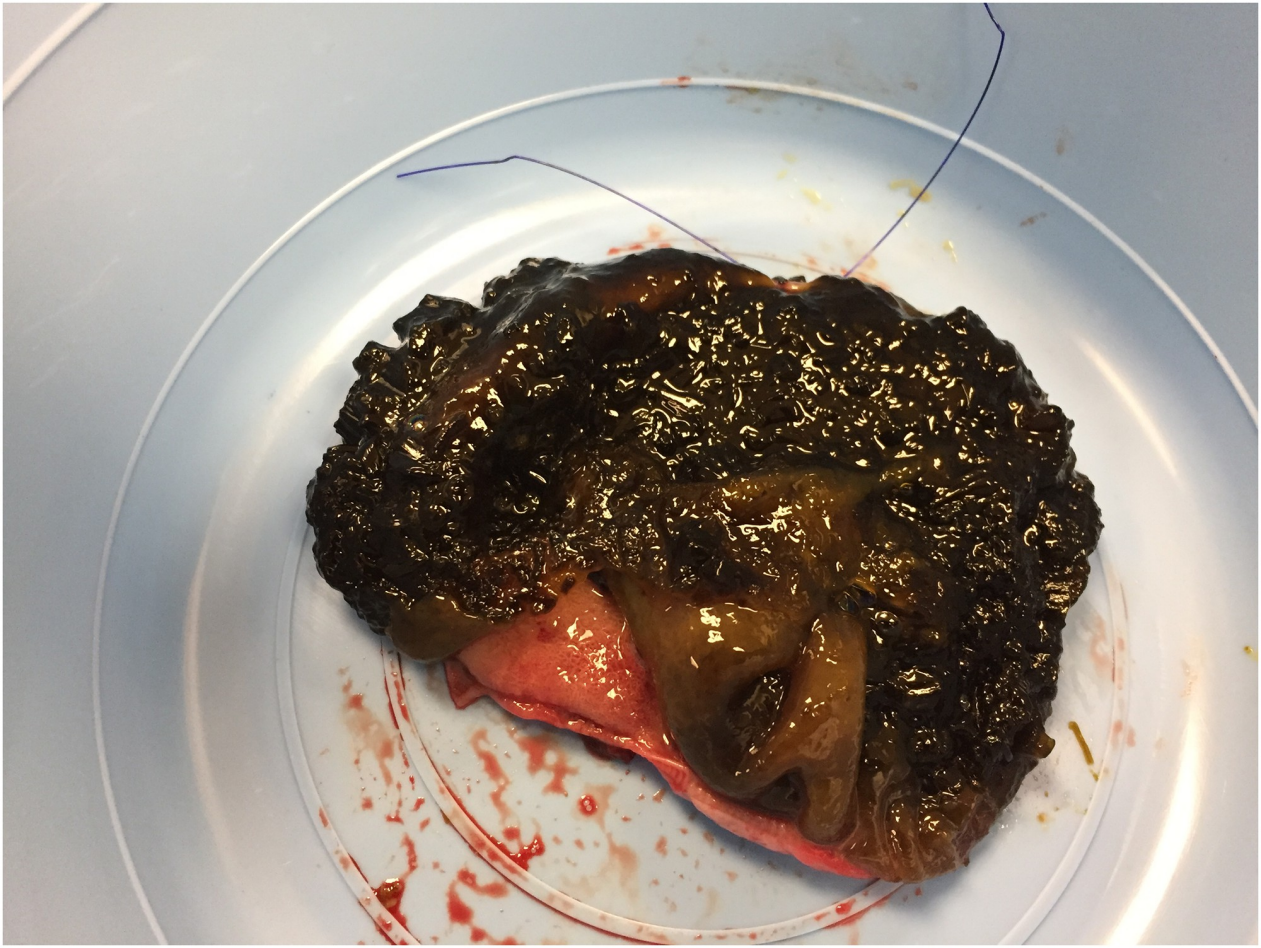
Interior appearance of a gallbladder mucocele after surgical removal from a dog. The gallbladder was opened lengthwise exposing the intraluminal mucus content which served as the source of sample used for 16S rRNA gene sequencing.

Samples of gallbladder mucus were collected from 13 dogs with mucocele formation (Fig 4). Represented breeds were Chihuahua (3 dogs), Shetland Sheepdog (2 dogs), American Cocker Spaniel, Bichon Frise, Hound, Jack Russell Terrier, Labrador Retriever, Maltese, Yorkshire Terrier, and mixed breed (1 dog each). Median age was 12 years (range, 4.8 to 16 years).

#### Microbiota composition of gallbladder mucocele mucus

After filtration of contaminating sequences and taxa with read counts ≤10 in all samples, mucus samples yielded a median of 5,915 reads (range, 0 to 144,164 reads) using the non-phenol extraction method and a median of 8,921 reads (range, 0 to 17,585,627 reads) using the phenol extraction method. A total of 109 unique ASVs were identified among mucocele mucus samples. Seventy-eight (71%) of these ASVs were not amplified from any negative extraction control samples (Table 2).

**Table 2:** Seventy eight amplicon sequence variants amplified from gallbladder mucus collected from 13 dogs with mucocele formation and not observed in extraction control samples that did not contain mucus.

Amplicon Sequence Variants	Dogs (n = 13)		Max % Abundance
	No.	%	
d__Bacteria;p__Firmicutes;c__Bacilli;o__Lactobacillales;f__Lactobacillaceae;g__Lactobacillus;s__Lactobacillus_brevis	4	30.8	52.45
d__Bacteria;p__Firmicutes;c__Clostridia;o__Peptostreptococcales-Tissierellales;f__Peptostreptococcales-Tissierellales;g__Peptoniphilus	4	30.8	8.08
d__Bacteria;p__Proteobacteria;c__Alphaproteobacteria;o__Rickettsiales;f__Mitochondria;g__Mitochondria	4	30.8	2.31
d__Bacteria;p__Firmicutes;c__Negativicutes;o__Veillonellales-Selenomonadales;f__Veillonellaceae;g__Veillonella;s__Veillonella_montpellierensis	3	23.1	8.55
d__Bacteria;p__Proteobacteria;c__Gammaproteobacteria;o__Burkholderiales;f__Burkholderiaceae;g__Ralstonia	3	23.1	1.99
d__Bacteria;p__Bacteroidota;c__Bacteroidia;o__Bacteroidales;f__Bacteroidaceae;g__Bacteroides	2	15.4	1.04
d__Bacteria;p__Bacteroidota;c__Bacteroidia;o__Bacteroidales;f__Tannerellaceae;g__Parabacteroides;s__Parabacteroides_distasonis	2	15.4	5.83
d__Bacteria;p__Bacteroidota;c__Bacteroidia;o__Flavobacteriales;f__Weeksellaceae;g__Chryseobacterium	2	15.4	2.09
d__Bacteria;p__Firmicutes;c__Bacilli;o__Bacillales;f__Bacillaceae;g__Bacillus;s__Bacillus_alcalophilus	2	15.4	0.60
d__Bacteria;p__Proteobacteria;c__Alphaproteobacteria;o__Rhizobiales;f__Rhizobiaceae;g__Ochrobactrum	2	15.4	60.00
d__Bacteria;p__Proteobacteria;c__Alphaproteobacteria;o__Sphingomonadales;f__Sphingomonadaceae;g__Novosphingobium	2	15.4	0.54
d__Bacteria;p__Proteobacteria;c__Gammaproteobacteria;o__Pseudomonadales;f__Moraxellaceae;g__Enhydrobacter	2	15.4	5.11
d__Bacteria;p__Bacteroidota;c__Bacteroidia;o__Bacteroidales;f__Bacteroidaceae;g__Bacteroides;s__Bacteroides_vulgatus	1	7.7	0.032
d__Bacteria;p__Proteobacteria;c__Alphaproteobacteria;o__Sphingomonadales;f__Sphingomonadaceae;g__Sphingomonas;s__Sphingomonas_koreensis	1	7.7	4.18
d__Bacteria;p__Actinobacteriota;c__Actinobacteria;o__Corynebacteriales;f__Corynebacteriaceae;g__Corynebacterium;s__Corynebacterium_aurimucosum	1	7.7	0.00052
d__Bacteria;p__Actinobacteriota;c__Actinobacteria;o__Corynebacteriales;f__Corynebacteriaceae;g__Corynebacterium;s__Corynebacterium_kroppenstedtii	1	7.7	0.26
d__Bacteria;p__Actinobacteriota;c__Actinobacteria;o__Corynebacteriales;f__Corynebacteriaceae;g__Turicella;s__uncultured_bacterium	1	7.7	1.36
d__Bacteria;p__Actinobacteriota;c__Actinobacteria;o__Corynebacteriales;f__Dietziaceae;g__Dietzia	1	7.7	4.91
d__Bacteria;p__Actinobacteriota;c__Actinobacteria;o__Corynebacteriales;f__Nocardiaceae;g__Rhodococcus;s__Rhodococcus_aerolatus	1	7.7	0.14
d__Bacteria;p__Actinobacteriota;c__Actinobacteria;o__Frankiales;f__Geodermatophilaceae;g__Blastococcus	1	7.7	0.56
d__Bacteria;p__Actinobacteriota;c__Actinobacteria;o__Frankiales;f__Geodermatophilaceae;g__Blastococcus;s__Blastococcus_aggregatus	1	7.7	3.80
d__Bacteria;p__Actinobacteriota;c__Actinobacteria;o__Micrococcales	1	7.7	0.20
d__Bacteria;p__Actinobacteriota;c__Actinobacteria;o__Micrococcales;f__Dermabacteraceae;g__Dermabacter	1	7.7	0.41
d__Bacteria;p__Actinobacteriota;c__Actinobacteria;o__Micrococcales;f__Dermacoccaceae	1	7.7	0.39
d__Bacteria;p__Actinobacteriota;c__Actinobacteria;o__Micrococcales;f__Dermacoccaceae;g__Dermacoccus;s__Dermacoccus_nishinomiyaensis	1	7.7	0.49
d__Bacteria;p__Actinobacteriota;c__Actinobacteria;o__Micrococcales;f__Microbacteriaceae;g__Leucobacter	1	7.7	0.74
d__Bacteria;p__Actinobacteriota;c__Actinobacteria;o__Micrococcales;f__Micrococcaceae;g__Kocuria	1	7.7	1.37
d__Bacteria;p__Actinobacteriota;c__Actinobacteria;o__Propionibacteriales;f__Nocardioidaceae	1	7.7	2.84
d__Bacteria;p__Actinobacteriota;c__Actinobacteria;o__Propionibacteriales;f__Propionibacteriaceae;g__Friedmanniella	1	7.7	0.66
d__Bacteria;p__Actinobacteriota;c__Actinobacteria;o__Propionibacteriales;f__Propionibacteriaceae;g__Propioniciclava	1	7.7	3.36
d__Bacteria;p__Actinobacteriota;c__Actinobacteria;o__Pseudonocardiales;f__Pseudonocardiaceae;g__Pseudonocardia	1	7.7	1.31
d__Bacteria;p__Actinobacteriota;c__Coriobacteriia;o__Coriobacteriales;f__Eggerthellaceae;g__Eggerthella;s__uncultured_bacterium	1	7.7	0.33
d__Bacteria;p__Bacteroidota;c__Bacteroidia;o__Bacteroidales;f__Muribaculaceae;g__Muribaculaceae;s__uncultured_bacterium	1	7.7	40.00
d__Bacteria;p__Bacteroidota;c__Bacteroidia;o__Bacteroidales;f__Tannerellaceae;g__Parabacteroides;s__Parabacteroides_merdae	1	7.7	0.24
d__Bacteria;p__Bacteroidota;c__Bacteroidia;o__Chitinophagales;f__Chitinophagaceae;g__Flaviaesturariibacter;s__uncultured_bacterium	1	7.7	0.21
d__Bacteria;p__Bacteroidota;c__Bacteroidia;o__Flavobacteriales;f__Flavobacteriaceae;g__Flavobacterium	1	7.7	0.21
d__Bacteria;p__Campilobacterota;c__Campylobacteria;o__Campylobacterales;f__Campylobacteraceae;g__Campylobacter;s__Campylobacter_ureolyticus	1	7.7	0.0035
d__Bacteria;p__Cyanobacteria;c__Cyanobacteriia;o__Chloroplast;f__Chloroplast;g__Chloroplast;s__uncultured_Streptophyta	1	7.7	1.14
d__Bacteria;p__Cyanobacteria;c__Cyanobacteriia;o__Cyanobacteriales;f__Chroococcidiopsaceae	1	7.7	0.35
d__Bacteria;p__Cyanobacteria;c__Cyanobacteriia;o__Cyanobacteriales;f__Chroococcidiopsaceae;g__Chroococcidiopsis_SAG_2023;s__uncultured_cyanobacterium	1	7.7	21.01
d__Bacteria;p__Firmicutes;c__Bacilli;o__Bacillales;f__Bacillaceae;g__Bacillus;s__Bacillus_halodurans	1	7.7	7.33
d__Bacteria;p__Firmicutes;c__Bacilli;o__Erysipelotrichales;f__Erysipelatoclostridiaceae;g__Erysipelatoclostridium	1	7.7	0.13
d__Bacteria;p__Firmicutes;c__Bacilli;o__Lactobacillales;f__Aerococcaceae;g__Abiotrophia;s__uncultured_bacterium	1	7.7	2.73
d__Bacteria;p__Firmicutes;c__Bacilli;o__Lactobacillales;f__Aerococcaceae;g__Aerococcus	1	7.7	0.81
d__Bacteria;p__Firmicutes;c__Bacilli;o__Lactobacillales;f__Enterococcaceae;g__Enterococcus;s__Enterococcus_cecorum	1	7.7	2.86
d__Bacteria;p__Firmicutes;c__Bacilli;o__Lactobacillales;f__Vagococcaceae;g__Vagococcus	1	7.7	3.14
d__Bacteria;p__Firmicutes;c__Bacilli;o__Staphylococcales;f__Staphylococcaceae;g__Salinicoccus	1	7.7	1.95
d__Bacteria;p__Firmicutes;c__Bacilli;o__Staphylococcales;f__Staphylococcaceae;g__Salinicoccus;s__uncultured_bacterium	1	7.7	1.35
d__Bacteria;p__Firmicutes;c__Clostridia;o__Lachnospirales;f__Lachnospiraceae;g__uncultured;s__uncultured_Eubacterium	1	7.7	4.27
d__Bacteria;p__Firmicutes;c__Clostridia;o__Peptostreptococcales-Tissierellales;f__Peptostreptococcales-Tissierellales;g__Anaerococcus;s__Anaerococcus_hydrogenalis	1	7.7	1.14
d__Bacteria;p__Firmicutes;c__Clostridia;o__Peptostreptococcales-Tissierellales;f__Peptostreptococcales-Tissierellales;g__Fenollaria;s__uncultured_bacterium	1	7.7	0.36
d__Bacteria;p__Firmicutes;c__Desulfitobacteriia;o__Desulfitobacteriales;f__Desulfitobacteriaceae;g__Desulfosporosinus;s__uncultured_bacterium	1	7.7	1.93
d__Bacteria;p__Myxococcota;c__Polyangia;o__Polyangiales;f__BIrii41;g__BIrii41	1	7.7	2.09
d__Bacteria;p__Patescibacteria;c__Saccharimonadia;o__Saccharimonadales;f__Saccharimonadaceae;g__Saccharimonadaceae	1	7.7	1.62
d__Bacteria;p__Planctomycetota;c__Planctomycetes;o__Pirellulales;f__Pirellulaceae;g__uncultured	1	7.7	0.41
d__Bacteria;p__Proteobacteria;c__Alphaproteobacteria;o__Reyranellales;f__Reyranellaceae;g__Reyranella	1	7.7	0.94
d__Bacteria;p__Proteobacteria;c__Alphaproteobacteria;o__Rhodobacterales;f__Rhodobacteraceae;g__Paracoccus	1	7.7	8.41
d__Bacteria;p__Proteobacteria;c__Alphaproteobacteria;o__Rickettsiales;f__Mitochondria;g__Mitochondria;s__Clonostachys_rosea	1	7.7	0.35
d__Bacteria;p__Proteobacteria;c__Alphaproteobacteria;o__Rickettsiales;f__Mitochondria;g__Mitochondria;s__Rhynchosporium_secalis	1	7.7	0.22
d__Bacteria;p__Proteobacteria;c__Alphaproteobacteria;o__Rickettsiales;f__Mitochondria;g__Mitochondria;s__Sclerotinia_borealis	1	7.7	2.02
d__Bacteria;p__Proteobacteria;c__Gammaproteobacteria;o__Burkholderiales;f__Alcaligenaceae	1	7.7	1.10
d__Bacteria;p__Proteobacteria;c__Gammaproteobacteria;o__Burkholderiales;f__Alcaligenaceae;g__Achromobacter	1	7.7	0.0063
d__Bacteria;p__Proteobacteria;c__Gammaproteobacteria;o__Burkholderiales;f__Comamonadaceae;g__Delftia	1	7.7	0.47
d__Bacteria;p__Proteobacteria;c__Gammaproteobacteria;o__Burkholderiales;f__Comamonadaceae;g__Polaromonas	1	7.7	1.19
d__Bacteria;p__Proteobacteria;c__Gammaproteobacteria;o__Burkholderiales;f__Neisseriaceae;g__uncultured;s__uncultured_bacterium	1	7.7	2.22
d__Bacteria;p__Proteobacteria;c__Gammaproteobacteria;o__Burkholderiales;f__Oxalobacteraceae;g__Massilia	1	7.7	0.079
d__Bacteria;p__Proteobacteria;c__Gammaproteobacteria;o__Burkholderiales;f__Oxalobacteraceae;g__Undibacterium	1	7.7	0.45
d__Bacteria;p__Proteobacteria;c__Gammaproteobacteria;o__Cellvibrionales;f__Cellvibrionaceae;g__Cellvibrio	1	7.7	2.96
d__Bacteria;p__Proteobacteria;c__Gammaproteobacteria;o__Enterobacterales	1	7.7	0.00048
d__Bacteria;p__Proteobacteria;c__Gammaproteobacteria;o__JG36-TzT-191;f__JG36-TzT-191;g__JG36-TzT-191;s__uncultured_proteobacterium	1	7.7	0.78
d__Bacteria;p__Proteobacteria;c__Gammaproteobacteria;o__Pasteurellales;f__Pasteurellaceae;g__Aggregatibacter	1	7.7	0.73
d__Bacteria;p__Proteobacteria;c__Gammaproteobacteria;o__Pasteurellales;f__Pasteurellaceae;g__Haemophilus	1	7.7	0.000068
d__Bacteria;p__Proteobacteria;c__Gammaproteobacteria;o__Pseudomonadales;f__Moraxellaceae;g__Acinetobacter;s__Acinetobacter_radioresistens	1	7.7	1.89
d__Bacteria;p__Proteobacteria;c__Gammaproteobacteria;o__Steroidobacterales;f__Steroidobacteraceae;g__Steroidobacter	1	7.7	1.29
d__Bacteria;p__Proteobacteria;c__Gammaproteobacteria;o__Xanthomonadales;f__Xanthomonadaceae;g__Stenotrophomonas;s__Stenotrophomonas_nitritireducens	1	7.7	0.54
d__Bacteria;p__Proteobacteria;c__Gammaproteobacteria;o__Xanthomonadales;f__Xanthomonadaceae;g__Stenotrophomonas;s__Stenotrophomonas_rhizophila	1	7.7	3.30
d__Bacteria;p__Verrucomicrobiota;c__Verrucomicrobiae;o__Pedosphaerales;f__Pedosphaeraceae;g__ADurb.Bin063-1;s__uncultured_bacterium	1	7.7	2.45
d__Bacteria;p__Verrucomicrobiota;c__Verrucomicrobiae;o__Verrucomicrobiales;f__Verrucomicrobiaceae;g__uncultured;s__uncultured_bacterium	1	7.7	0.12

The remaining 31 ASVs were also amplified from ≥ 1 negative extraction control samples and were retained in the analysis because their read count in mucus exceeded the maximum count observed in any negative extraction controls (S4 Table).

The majority of ASVs in Table 2 (66/78; 85%) were observed in only a single mucus sample. The majority of ASVs 56/78 (72%) were also different in identity to ASVs observed in control bile samples. Three taxa were shared most commonly among mucocele mucus samples including *Lactobacillus brevis* (4 dogs; range, 10 to 4395 reads) and members of the genus *Peptoniphilus* (4 dogs; range, 6 to 677 reads) and *Mitochondria* (4 dogs; range, 17 to 138 reads). In no dogs was the presence of these 3 taxa confirmed in the sample using both extraction methods.

The number of unique ASVs per mucus sample ranged from 0 to 23 (median, 4) using the non-phenol extraction method and 0 to 19 (median, 5) using the phenol extraction method (Fig 3B and S5 Table). Only 4 of the 13 mucus samples had ≥ 1 ASV (range, 1 to 2 ASV) amplified from the same sample using both extraction methods. The most common ASV to be confirmed by both extraction methods was the genus *Escherichia coli/Shigella* (samples 13 and 26).

#### Conventional detection of bacteria

**Fig 5:**
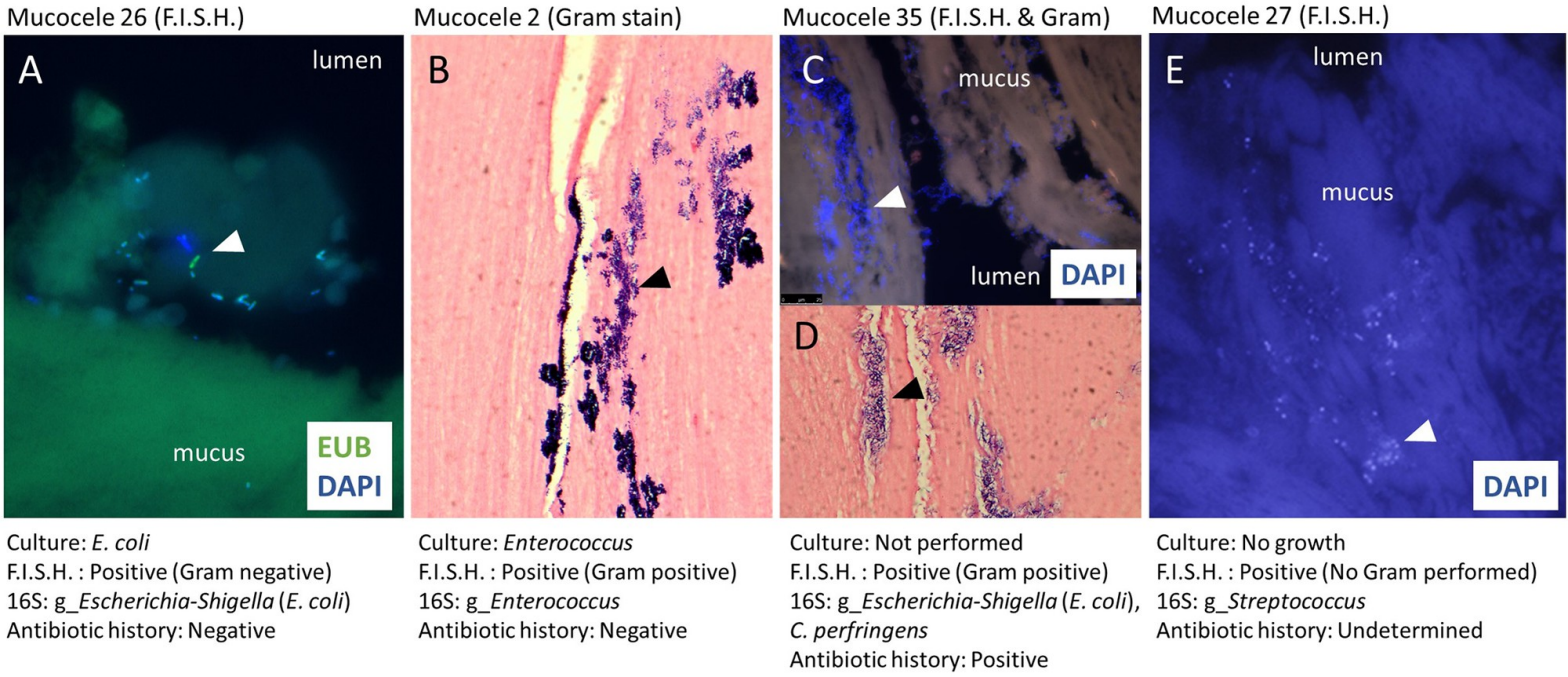
Results of fluorescence in-situ hybridization (F.I.S.H.) for eubacteria and Gram stain of gallbladder mucosa from 4 dogs with mucocele formation and concurrent findings of conventional culture and 16S rRNA gene sequencing of mucus content. Bacteria are indicated by closed arrowhead and visualized by means of positive hybridization with Eub 338—6FAM (green; panel A), Gram stain (panel B and D), or DAPI staining of bacterial nucleic acid (blue; panel A, C, and E).

Aerobic and anaerobic culture was performed on gallbladder mucus samples from 12/13 dogs with mucocele formation. Bacterial growth was identified in 4/12 (33%) mucus samples and included *E*. *coli* (3 dogs) and *Enterococcus* spp. (1 dog). Parallel results of 16S rRNA gene amplicon sequencing confirmed presence of the cultured bacterial isolate in 3/4 culture positive dogs and bacteria were observed by FISH in 2/3 culture positive dogs that had FISH performed (Fig 5 and S5 Table). For the remaining 8 culture negative and 1 uncultured sample, 2 had sequencing results consistent with presence of common and cultivable bacteria including *E*. *coli*, and *C*. *perfringens* (sample 35; FISH-positive) or *Streptococcus* (sample 27; FISH-positive)(Fig 5 and S5 Table). The remaining samples were dominated by unassigned bacteria or a member of the genus *Geobacillus*. Identified as a contaminating ASV, *Geobacillus* was observed in both control bile samples and mucocele mucus samples and exclusively in those extracted using the no phenol protocol (S3 and S5 Tables).

### Alpha and beta diversity metrics

**Fig 6:**
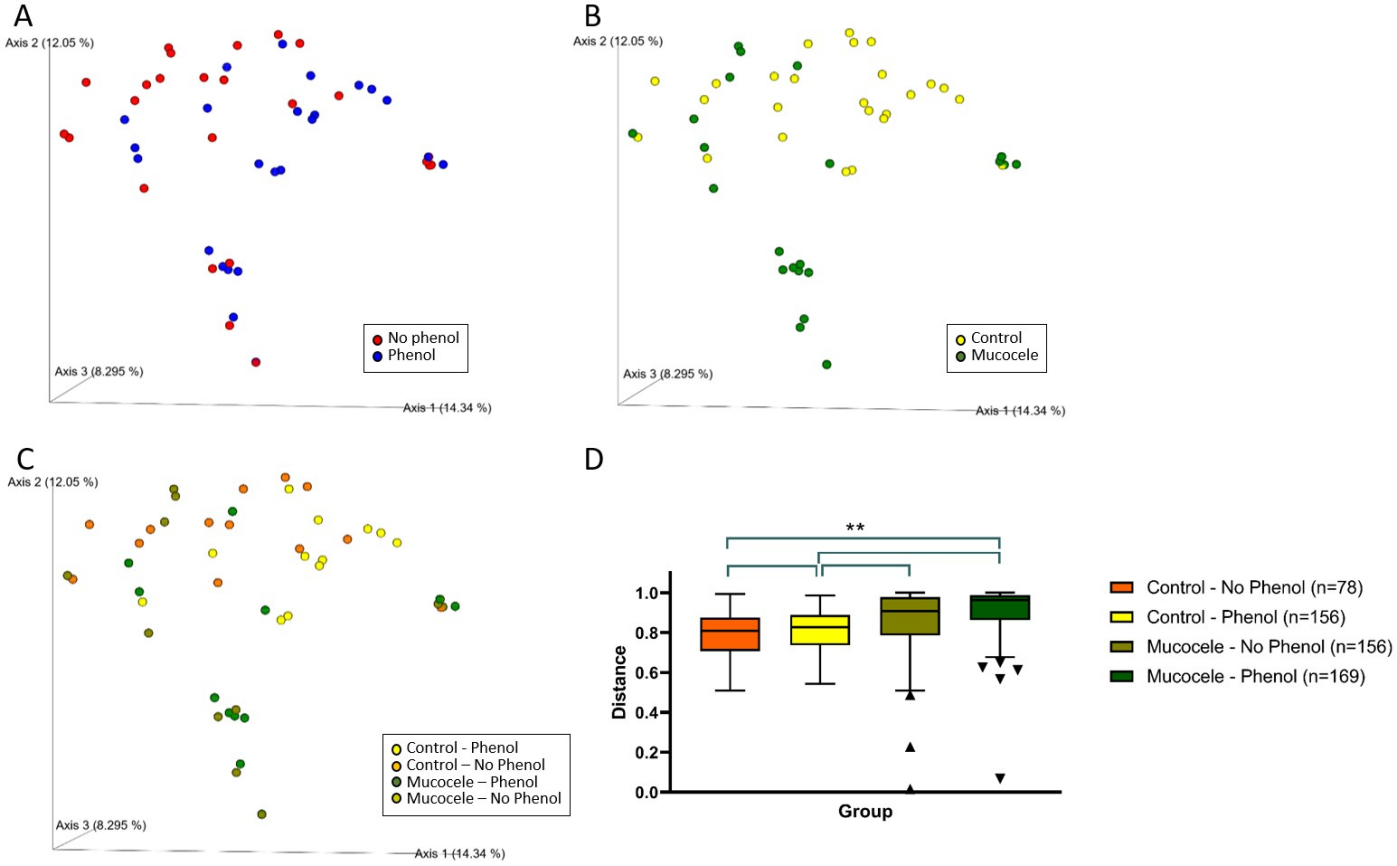
Beta diversity of amplicon sequence variants in gallbladder bile from 13 healthy dogs and gallbladder mucus from 13 dogs with mucocele formation each having DNA extracted using two different methods. Bray-Curtis principal coordinates analysis plots showing clustering of microbial communities based on isolation method (Panel A), disease condition (Panel B), and disease condition by isolation method (Panel C). Axis 1, 8.295%; Axis 2, 12.05%; Axis 3, 14.34%. Group significance plot comparing distances from Control—No Phenol (Panel D). Brackets represent significant differences between groups (PERMANOVA **q < 0.01). Box and Tukey whiskers represent interquartile range (IQR) and ± 1.5×IQR, respectively.

At a rarefaction depth of 1,000 reads, there were no significant differences in alpha diversity measures of evenness (Shannon), diversity (Faith), or observed number of ASV between samples of gallbladder bile from healthy dogs and samples of mucus from the gallbladder of dogs with mucocele formation. Both weighted and unweighted measures of beta diversity identified a significant difference in composition of ASV between control and mucocele gallbladder samples (Bray-Curtis PERMANOVA q = 0.002). Extraction method was observed to significantly affect only non-phylogenetic based differences in beta diversity (Bray-Curtis PERMANOVA q = 0.015)(Fig 6).

## Discussion

This study is the first to characterize the culture-independent microbiota composition of bile in healthy dogs using next-generation sequencing. Comparable studies in healthy people are lacking because of the invasive nature of bile sampling and are therefore limited to bile collected from patients with biliary disease [2–11], from the gallbladder of transplanted livers [1, 9], or using inherently contaminated approaches such as during endoscopic retrograde catheterization of the common bile duct [2, 5, 8, 10, 11]. Few of these studies report sequencing results of control extractions or describe how identified reagent and laboratory DNA contaminants were supervised during data analysis [6]. In our study, 67% of taxa identified as contaminants by amplification from ≥ 1 negative control extraction were also observed in results of 16S rRNA gene amplicon sequencing of samples of gallbladder bile and mucus from dogs. This observation points out how significantly contaminating ASVs can contribute to the results of sequencing analysis when performed on samples with low bacterial biomass.

While contaminating ASVs were common in sequence data from bile samples, the majority of ASVs identified in bile from healthy dogs did not overlap with ASV observed in negative control extractions. Nonetheless, most of these ASVs were identified in only 1 or 2 bile samples. These findings do not support the existence of a core bile microbiota. Absence of a core microbiota is also supported by concurrent negative results of aerobic and anaerobic bacterial culture, cytological examination of bile, and eubacterial FISH performed on gallbladder mucosa. What remains unclear is whether these ASVs identified in bile from healthy dogs reflects genuinely endogenous bacterial DNA or an unaccounted for source of additional contamination. If genuine, our findings suggest that genesis of this microbiota would have to be unique to each individual and seemingly random. Notably, within each bile sample, amplification of individual taxa was not reproducible when comparing results of the 2 different DNA isolation methods. This lack of reproducibility is considered a hallmark of 16S rRNA sequence contamination [36]. It is important to consider that extraction controls alone are insufficient for detection of all points of bacterial DNA contamination [51]. For example, contamination introduced during sample collection can be accounted for by sequencing of technical controls that mimic the collection process and materials but without obtaining the actual sample. Our study did not include technical controls, and had we done so, we submit it is likely that many of the ASVs observed in these bile samples would overlap in identity with contaminants. In particular, *Stenotrophomonas*, *Novosphingobium*, *Bacillus halodurans*, and *Massilia* are worthy of brief discussion as they were observed in samples from several healthy control dogs. Each of these taxa were amplified at a low read count, were inconsistently validated by recovery using both extraction methods, and are well-recognized as either environmental extremophiles or water and soil-associated bacterial contaminants of sequencing studies [35, 52–55]. Although the post-mortem collection method used for control bile samples in our study did not appear to have a significant influence on the results, future studies may further minimize contamination by performing a sterile preparation of the abdominal skin followed by either ultrasound-guided bile aspiration or laparotomy using sterile gloves.

Unexpectedly to us, 16S rRNA gene sequences for well-recognized biliary pathogens such as *Enterococcus* spp. and *E*.*coli-Shigella* were common and abundant contaminants of the extraction materials and reagents. This presents a major limitation to the use of 16S sequencing approaches for distinguishing infection versus contamination with these bacteria or their DNA in bile samples. In some instances in this study, the presence of these bacteria could have been missed simply because low copy number of 16S rRNA gene sequences fell within the expectations of background contamination. A single control dog in the study had a large abundance of *E*. *coli-Shigella* and *Streptococcus* sp. sequences in bile consistent with likely infection. It is possible that this dog was a false-negative for bacterial culture or the sample had an unusually high level of contamination. Because the history of apparently healthy shelter dogs was unknown, occult bile infection of this dog is possible.

The second aim of our study was to determine whether the gallbladder of dogs with mucocele formation is systematically colonized by any uncultivable bacterial taxa that could possibly play a role in disease pathogenesis. To date, evidence suggesting a role for bacteria in the primary pathogenesis of gallbladder mucocele formation in dogs has been largely circumstantial. Retrospective studies identify that a median of 13% of dogs with mucocele formation have positive bacterial culture results of gallbladder content [22, 23, 25, 27, 28, 31–34]. In our study, positive culture results for either *E*. *coli* or *Enterococcus* spp. were obtained for 30% of gallbladder mucus samples. *Escherichia coli* and *Enterococcus* spp. are the most common bacterial causes of cholecystitis in dogs [13–21] and the most common isolates identified in the gallbladder of dogs with mucocele formation [22, 23, 25, 27, 28, 31–34]. The presence of visible bacteria was confirmed by FISH performed on gallbladder mucosa from 4 dogs (30%), which is similar in prevalence to another study reporting FISH results on gallbladder tissue from dogs with mucocele formation [56]. In this study, bacteria observed by FISH were concordant with either a positive culture result or abundant 16S rRNA gene amplification of a culturable species of enteric bacteria (i.e. *E*. *coli*, *Clostridium perfringens*, and *Streptococcus* spp.) from gallbladder mucus. In each positive instance, FISH demonstrated the bacteria as largely confined to superficial layers of the gallbladder mucus. Collectively, these findings support prior observations that gallbladder mucoceles can become infected by common enteric bacteria [22, 23, 25, 27, 28, 31–34]. These infections may ascend from the intestinal tract, arise secondary to impaired ability of the liver to clear bacteria from the portal circulation, or result from transient or physiologic bacteremia that finds a nidus in which to grow on the mucocele or diseased biliary epithelium.

A range from 0 to 23 different uncultured ASVs were identified by 16S rRNA gene amplicon sequencing of mucus samples from dogs with gallbladder mucocele formation. Roughly 28% of these ASVs were also amplified from negative extraction controls and therefore consistent with documented reagent and laboratory DNA contaminants. High within-sample abundance was observed for some ASVs such as *Geobacillus*, a recognized contaminant [57, 58] which was amplified exclusively from samples extracted using the no phenol method. Other highly abundant ASVs amplified from mucocele mucus including *Ochrobactrum* [35], *Sphingobium* [35, 57], and *Bacillus* [35, 53, 57] are well-documented DNA contaminants of extraction kits and reagents. Akin to our observations in healthy dog bile, few ASVs were shared by more than 1 mucus sample and none of the shared ASVs had their presence confirmed in any sample by both DNA extraction methods. Among these, *Peptoniphilus* and *Lactobacillus brevis* were each identified in 4 dogs. *Peptoniphilus* is recognized as a contaminant [57], however we could not locate any prior reports of contamination by *Lactobacillus brevis*. Amplification of *Lactobacillus brevis* was not restricted to one extraction method, however failure to simultaneously demonstrate this ASV in any mucus sample by both extraction methods is a trademark of DNA contamination. Collectively, these findings do not support the existence of a core mucus microbiome in the gallbladder of dogs with mucocele formation nor identify any taxa that are systematically associated with this disease.

It is remarkable that 72% of the ASVs identified in the gallbladder mucus of dogs with mucocele formation were different in identity to ASVs observed in control bile samples. It is possible that this reflects predilection of the gallbladder for different consortia of bacteria in the setting of mucocele formation. But it is reasonable to consider that the difference could be attributed to differences in contaminants introduced during the sample collection process. Control bile samples were collected with a syringe and needle directly from the gallbladder, while mucus samples were collected with surgical instruments after removal of the gallbladder and incision through the gallbladder wall. Limitations of our study are lack of inclusion of technical controls that would have enabled confirmation of contaminants introduced during sample collection. Other limitations are potential confounding effects of antibiotic administration in some dogs with gallbladder mucocele formation which is an unavoidable circumstance when studying clinical patients. A history of antibiotic administration was not associated with any obvious discordance between culture, FISH, and 16S sequencing results for dogs in the study.

In conclusion, this study does not support the existence of a core microbiome in the bile of dogs nor any systematic influence of uncultured bacteria on pathogenesis of gallbladder mucocele formation. Our elected approach of using 2 different DNA extraction methods for parallel 16S rRNA gene amplicon sequencing of each sample and sequencing of negative control extractions enabled identification of authenticated and putative contaminating DNA sequences that was essential to interpretation of these findings.

## Supporting information

S1 Table Contaminating sequences observed in extraction control samples under each of the 4 extraction conditions. Data represent the average number of reads and average % abundance of 3 replicates for each condition.
(DOCX)

S2 Table Twenty seven amplicon sequence variants amplified from bile collected from 13 apparently healthy adult dogs that were also amplified from ≥ 1 negative extraction control sample but at lower read counts than observed in bile. (DOCX)

S3 Table Amplicon sequence variants (ASV) identified by 16S rRNA gene amplification and Illumina-based sequencing from individual samples of bile collected from 13 healthy dogs and extracted using 2 commercial approaches. Results of conventional cytology, aerobic and anaerobic bacterial culture, and eubacterial fluorescence in-situ hybridization (FISH) are also shown for each sample.
(DOCX)

S4 Table Thirty one amplicon sequence variants amplified from gallbladder mucus collected from 13 dogs diagnosed with mucocele formation that were also amplified from ≥ 1 negative extraction control sample but at lower abundance than observed in mucus. (DOCX)

S5 Table Amplicon sequence variants (ASV) identified by 16S rRNA gene amplification and Illumina-based sequencing from individual samples of gallbladder mucus collected from 13 dogs with mucocele formation and extracted using 2 commercial approaches. Results of aerobic and anaerobic bacterial culture, and eubacterial fluorescence in-situ hybridization (FISH) are also shown for each sample.
(DOCX)
