# Supplementary material for: Gallbladder microbiota in healthy dogs and dogs with mucocele formation
Source: PLoS One. 2023 Feb 10;18(2):e0281432. doi: 10.1371/journal.pone.0281432 (PMC9916591; doi:10.1371/journal.pone.0281432)
Supplement: S1 Table — Data represent the average number of reads and average % abundance of 3 replicates for each condition. (DOCX) [file pone.0281432.s001.docx]

| **Classification** | | | **No swab** | | | | **Swab** | | | |
| --- | --- | --- | --- | --- | --- | --- | --- | --- | --- | --- |
|  |  |  | **Phenol** | | **No Phenol** | | **Phenol** | | **No Phenol** | |
| **Domain/Phylum** | **Family** | **Genus_and species** | **# reads** | **% abundance** | **# reads** | **% abundance** | **# reads** | **% abundance** | **# reads** | **% abundance** |
| Unassigned |  |  | 287 | 0.32 | 34 | 0.46 | 226 | 0.86 | 39 | 0.41 |
| Bacteria |  |  |  |  |  |  | 11 | 0.041 | 1 | 0.0069 |
| Actinobacteriota | Iamiaceae | Iamia | 4 | 0.0041 |  |  |  |  |  |  |
|  | Actinomycetaceae | Actinomyces Schaalia odontolytica |  |  |  |  |  |  | 4 | 0.038 |
|  | Bifidobacteriaceae | Bifidobacterium |  |  |  |  | 14 | 0.055 |  |  |
|  | Corynebacteriaceae | Corynebacterium | 964 | 1.08 | 65 | 0.88 | 2737 | 10.41 | 96 | 0.99 |
|  |  | Lawsonella uncultured bacterium | 235 | 0.26 |  |  | 356 | 1.36 |  |  |
|  | Mycobacteriaceae | Mycobacterium |  |  |  |  | 156 | 0.59 | 146 | 1.50 |
|  | Nocardiaceae | Rhodococcus | 18 | 0.020 |  |  |  |  |  |  |
|  | Brevibacteriaceae | Brevibacterium uncultured bacterium |  |  |  |  | 181 | 0.69 |  |  |
|  | Dermacoccaceae | Dermacoccus |  |  |  |  |  |  | 20 | 0.21 |
|  | Microbacteriaceae | Agromyces |  |  |  |  |  |  | 2 | 0.021 |
|  |  | Curtobacterium |  |  |  |  | 7 | 0.025 |  |  |
|  |  | Leifsonia | 4 | 0.0049 |  |  |  |  |  |  |
|  |  | Microbacterium | 163 | 0.18 |  |  |  |  |  |  |
|  | Micrococcaceae | Micrococcus | 10 | 0.011 |  |  | 40 | 0.15 |  |  |
|  |  | Nesterenkonia Nesterenkonia sp. | 107 | 0.12 |  |  |  |  |  |  |
|  | Sanguibacteraceae | Sanguibacte |  |  |  |  |  |  | 19 | 0.20 |
|  | Micromonosporaceae | Micromonospora |  |  |  |  |  |  | 64 | 0.66 |
|  | Propionibacteriaceae | Cutibacterium | 1012 | 1.14 | 357 | 4.82 | 8935 | 33.99 | 139 | 1.43 |
|  |  | Cutibacterium granulosum | 45 | 0.051 |  |  | 82 | 0.31 |  |  |
|  |  | Microlunatus | 37 | 0.042 |  |  |  |  |  |  |
|  | Pseudonocardiaceae | Saccharothrix |  |  |  |  | 11 | 0.042 |  |  |
| Armatimonadota | Fimbriimonadaceae | Fimbriimonadaceae |  |  |  |  |  |  | 3 | 0.034 |
| Bacteroidota | Prevotellaceae | Alloprevotella uncultured Bacteroidetes |  |  |  |  | 8 | 0.029 |  |  |
|  |  | Prevotella aurantiaca |  |  |  |  | 7 | 0.025 |  |  |
|  | Spirosomaceae | Flectobacillus |  |  | 89 | 1.20 |  |  |  |  |
|  | Flavobacteriaceae | Flavobacterium Cytophaga sp. |  |  |  |  |  |  | 36 | 0.37 |
|  | Weeksellaceae |  |  |  |  |  |  |  | 35 | 0.36 |
|  | Weeksellaceae | Cloacibacterium | 19 | 0.021 |  |  |  |  |  |  |
|  | env.OPS_17 | env.OPS_17 |  |  |  |  |  |  | 70 | 0.73 |
| Cyanobacteria | Chloroplast | Chloroplast | 69 | 0.078 |  |  |  |  | 97 | 1.00 |
|  | Obscuribacteraceae | Candidatus Obscuribacter uncultured bacterium |  |  | 99 | 1.34 | 70 | 0.27 |  |  |
| Deinococcota | Deinococcaceae | Deinococcus |  |  |  |  |  |  | 1 | 0.0069 |
|  | Thermaceae | Thermus |  |  |  |  |  |  | 124 | 1.28 |
| Firmicutes | Bacillaceae |  | 6031 | 6.79 |  |  | 212 | 0.81 |  |  |
|  | Bacillaceae | Anaerobacillus |  |  |  |  | 22 | 0.085 |  |  |
|  |  | Anoxybacillus | 50409 | 56.75 | 230 | 3.10 | 2022 | 7.69 |  |  |
|  |  | Bacillus | 176 | 0.20 | 117 | 1.58 | 252 | 0.96 | 167 | 1.72 |
|  |  | Geobacillus |  |  | 661 | 8.92 |  |  | 627 | 6.47 |
|  | Planococcaceae |  |  |  | 3 | 0.041 |  |  |  |  |
|  | Brevibacillaceae | Brevibacillus | 6 | 0.0068 |  |  |  |  |  |  |
|  |  | Brevibacillus_thermoruber | 60 | 0.067 | 24 | 0.33 |  |  |  |  |
|  | Erysipelotrichaceae | Ileibacterium valens |  |  | 44 | 0.60 |  |  | 118 | 1.21 |
|  |  | Turicibacter |  |  |  |  |  |  | 133 | 1.37 |
|  | Enterococcaceae | Enterococcus | 5582 | 6.28 | 1619 | 21.87 | 1689 | 6.42 | 381 | 3.93 |
|  | Lactobacillaceae | Lactobacillus |  |  |  |  | 201 | 0.76 | 22 | 0.23 |
|  |  | Lactobacillus fermentum | 132 | 0.15 |  |  | 67 | 0.25 | 7 | 0.069 |
|  |  | Lactobacillus rhamnosus |  |  |  |  | 24 | 0.090 |  |  |
|  |  | Pediococcus |  |  |  |  |  |  | 32 | 0.33 |
|  | Leuconostocaceae | Leuconostoc |  |  | 3 | 0.036 |  |  | 43 | 0.44 |
|  | Listeriaceae | Listeria |  |  |  |  | 60 | 0.23 | 5 | 0.048 |
|  | Streptococcaceae | Lactococcus |  |  | 68 | 0.92 |  |  | 205 | 2.12 |
|  |  | Lactococcus lactis |  |  | 143 | 1.93 |  |  | 19 | 0.20 |
|  |  | Streptococcus | 17 | 0.020 | 6 | 0.081 | 22 | 0.082 | 2951 | 30.45 |
|  |  | Streptococcus salivarius | 7 | 0.0075 |  |  |  |  | 70 | 0.72 |
|  | Paenibacillaceae | Paenibacillus | 75 | 0.084 |  |  |  |  |  |  |
|  |  | Paenibacillus alginolyticus | 546 | 0.61 |  |  |  |  |  |  |
|  | Staphylococcaceae | Macrococcus |  |  | 126 | 1.70 |  |  |  |  |
|  |  | Staphylococcus | 1126 | 1.27 | 294 | 3.98 | 4816 | 18.32 | 253 | 2.61 |
|  | Clostridiaceae | Clostridium perfringens |  |  | 10 | 0.14 |  |  |  |  |
|  | Peptostreptococcales-Tissierellales | Anaerococcus | 13 | 0.014 |  |  | 346 | 1.32 |  |  |
|  |  | Anaerococcus uncultured organism |  |  |  |  | 271 | 1.03 |  |  |
|  |  | Finegoldia uncultured bacterium |  |  |  |  | 126 | 0.48 |  |  |
|  | Desulfotomaculales | Desulfohalotomaculum Desulfotomaculum sp. |  |  | 1 | 0.014 |  |  |  |  |
| Fusobacteriota | Leptotrichiaceae | Leptotrichia | 71 | 0.080 |  |  |  |  |  |  |
|  |  | Leptotrichia buccalis | 11 | 0.013 |  |  |  |  |  |  |
| Patescibacteria | Saccharimonadales | Saccharimonadales uncultured cyanobacterium |  |  |  |  |  |  | 5 | 0.052 |
| Alphaproteobacteria | Acetobacteraceae | Craurococcus-Caldovatus Craurococcus roseus |  |  |  |  | 9 | 0.036 |  |  |
|  | Caulobacteraceae | Brevundimonas | 56 | 0.063 |  |  |  |  |  |  |
|  | Paracaedibacteraceae | Candidatus Finniella uncultured bacterium |  |  |  |  |  |  | 4 | 0.041 |
|  | Beijerinckiaceae | Bosea | 203 | 0.23 |  |  |  |  |  |  |
|  |  | Methylobacterium-Methylorubrum | 112 | 0.13 |  |  |  |  |  |  |
|  | Rhizobiaceae |  | 216 | 0.24 |  |  |  |  |  |  |
|  | Rhizobiaceae | Allorhizobium-Neorhizobium-Pararhizobium-Rhizobium |  |  | 37 | 0.50 |  |  |  |  |
|  |  | Mesorhizobium | 194 | 0.22 |  |  |  |  |  |  |
|  | Xanthobacteraceae |  | 9 | 0.011 |  |  |  |  |  |  |
|  | Xanthobacteraceae | Afipia uncultured bacterium | 68 | 0.077 |  |  |  |  |  |  |
|  |  | Bradyrhizobium | 91 | 0.10 |  |  |  |  |  |  |
|  | Rhodobacteraceae | Rubellimicrobium |  |  |  |  | 5 | 0.020 |  |  |
|  | Mitochondria | Mitochondria Verticillium nonalfalfae |  |  |  |  |  |  | 5 | 0.052 |
|  | Sphingomonadaceae | Qipengyuania |  |  |  |  |  |  | 56 | 0.58 |
|  |  | Sphingobium | 13397 | 15.08 | 15 | 0.20 | 140 | 0.53 | 55 | 0.57 |
|  |  | Sphingomonas | 536 | 0.60 |  |  |  |  | 1 | 0.0069 |
| Gammaproteobacteria | Aeromonadaceae | Aeromonas |  |  |  |  | 4 | 0.014 |  |  |
|  | Shewanellaceae | Shewanella |  |  | 71 | 0.95 |  |  |  |  |
|  | Burkholderiaceae | Burkholderia-Caballeronia-Paraburkholderia |  |  |  |  |  |  | 22 | 0.22 |
|  | Chromobacteriaceae | Vogesella uncultured bacterium | 11 | 0.013 |  |  |  |  |  |  |
|  | Comamonadaceae |  | 2650 | 2.98 | 18 | 0.25 | 262 | 1.00 | 7 | 0.076 |
|  | Comamonadaceae | Curvibacte |  |  |  |  |  |  | 8 | 0.086 |
|  |  | Schlegelella uncultured bacterium | 14 | 0.016 |  |  |  |  |  |  |
|  |  | Tepidimonas | 250 | 0.28 |  |  |  |  | 7 | 0.072 |
|  |  | Variovorax | 1247 | 1.40 |  |  |  |  | 134 | 1.38 |
|  | Oxalobacteraceae |  |  |  |  |  | 70 | 0.27 |  |  |
|  | Oxalobacteraceae | Herbaspirillum | 47 | 0.053 |  |  |  |  |  |  |
|  | Enterobacteriaceae |  | 235 | 0.26 |  |  | 230 | 0.87 | 81 | 0.84 |
|  | Enterobacteriaceae | Escherichia-Shigella | 1278 | 1.44 | 1979 | 26.73 | 687 | 2.61 | 1370 | 14.14 |
|  | Erwiniaceae | Pantoea |  |  |  |  | 33 | 0.13 |  |  |
|  | Morganellaceae | Morganella | 247 | 0.28 |  |  |  |  |  |  |
|  | Yersiniaceae |  | 33 | 0.037 | 92 | 1.24 | 420 | 1.60 |  |  |
|  | Halomonadaceae | Halomonas | 131 | 0.15 |  |  |  |  | 69 | 0.72 |
|  | Moraxellaceae | Acinetobacter |  |  | 240 | 3.25 | 480 | 1.83 | 867 | 8.95 |
|  |  | Perlucidibaca uncultured bacterium |  |  |  |  |  |  | 8 | 0.079 |
|  | Pseudomonadaceae | Pseudomonas | 555 | 0.62 | 937 | 12.65 | 927 | 3.53 | 1066 | 10.99 |
|  |  | Pseudomonas peli | 11 | 0.013 |  |  |  |  |  |  |
|  | Vibrionaceae | Vibrio |  |  | 22 | 0.29 |  |  |  |  |
| Eukaryota |  |  | 1 | 0.0011 |  |  |  |  |  |  |
| Basidiomycota | Malasseziaceae | Malassezia uncultured Basidiomycota |  |  |  |  | 51 | 0.19 |  |  |
