# Supplementary material for: Gallbladder microbiota in healthy dogs and dogs with mucocele formation
Source: PLoS One. 2023 Feb 10;18(2):e0281432. doi: 10.1371/journal.pone.0281432 (PMC9916591; doi:10.1371/journal.pone.0281432)
Supplement: S2 Table — (DOCX) [file pone.0281432.s002.docx]

**S2 Table** Twenty seven amplicon sequence variants amplified from bile collected from 13 apparently healthy adult dogs that were also amplified from ≥ 1 negative extraction control sample but at lower read counts than observed in bile.

| **Amplicon Sequence Variants** | **Dogs (n=13)** | | **Max % Abundance** |
| --- | --- | --- | --- |
|  | **No.** | **%** |  |
| d__Bacteria | 4 | 31 | 28 |
| d__Bacteria;p__Firmicutes;c__Bacilli;o__Bacillales;f__Bacillaceae;g__Geobacillus | 4 | 31 | 92 |
| d__Bacteria;p__Proteobacteria;c__Gammaproteobacteria;o__Burkholderiales;f__Comamonadaceae;g__Curvibacter | 4 | 31 | 8.9 |
| Unassigned | 3 | 23 | 87 |
| d__Bacteria;p__Cyanobacteria;c__Cyanobacteriia;o__Chloroplast;f__Chloroplast;g__Chloroplast | 3 | 23 | 4.8 |
| d__Bacteria;p__Firmicutes;c__Bacilli;o__Bacillales;f__Bacillaceae;g__Bacillus | 3 | 23 | 38 |
| d__Bacteria;p__Proteobacteria;c__Gammaproteobacteria;o__Enterobacterales;f__Enterobacteriaceae | 3 | 23 | 60 |
| d__Eukaryota | 3 | 23 | 1.2 |
| d__Bacteria;p__Firmicutes;c__Bacilli;o__Brevibacillales;f__Brevibacillaceae;g__Brevibacillus;s__Brevibacillus_thermoruber | 2 | 15 | 46 |
| d__Bacteria;p__Proteobacteria;c__Alphaproteobacteria;o__Paracaedibacterales;f__Paracaedibacteraceae;g__Candidatus_Finniella;s__uncultured_bacterium | 2 | 15 | 52 |
| d__Bacteria;p__Proteobacteria;c__Gammaproteobacteria;o__Aeromonadales;f__Aeromonadaceae;g__Aeromonas;__ | 2 | 15 | 1.2 |
| d__Bacteria;p__Proteobacteria;c__Gammaproteobacteria;o__Pseudomonadales;f__Pseudomonadaceae;g__Pseudomonas | 2 | 15 | 67 |
| d__Bacteria;p__Actinobacteriota;c__Actinobacteria;o__Corynebacteriales;f__Nocardiaceae;g__Rhodococcus | 1 | 8 | 33 |
| d__Bacteria;p__Actinobacteriota;c__Actinobacteria;o__Micrococcales;f__Micrococcaceae;g__Micrococcus | 1 | 8 | 12 |
| d__Bacteria;p__Bacteroidota;c__Bacteroidia;o__Cytophagales;f__Spirosomaceae;g__Flectobacillus;__ | 1 | 8 | 36 |
| d__Bacteria;p__Bacteroidota;c__Bacteroidia;o__Flavobacteriales;f__Flavobacteriaceae;g__Flavobacterium;s__Cytophaga_sp. | 1 | 8 | 5.4 |
| d__Bacteria;p__Bacteroidota;c__Bacteroidia;o__Flavobacteriales;f__Weeksellaceae;g__Cloacibacterium | 1 | 8 | 11 |
| d__Bacteria;p__Cyanobacteria;c__Vampirivibrionia;o__Obscuribacterales;f__Obscuribacteraceae;g__Candidatus_Obscuribacter;s__uncultured_bacterium | 1 | 8 | 13 |
| d__Bacteria;p__Firmicutes;c__Bacilli;o__Bacillales;f__Bacillaceae;g__Anaerobacillus | 1 | 8 | 14 |
| d__Bacteria;p__Firmicutes;c__Bacilli;o__Lactobacillales;f__Lactobacillaceae;g__Lactobacillus | 1 | 8 | 5.8 |
| d__Bacteria;p__Firmicutes;c__Bacilli;o__Lactobacillales;f__Listeriaceae;g__Listeria | 1 | 8 | 2.7 |
| d__Bacteria;p__Firmicutes;c__Bacilli;o__Lactobacillales;f__Streptococcaceae;g__Lactococcus;s__Lactococcus_lactis | 1 | 8 | 26 |
| d__Bacteria;p__Firmicutes;c__Bacilli;o__Lactobacillales;f__Streptococcaceae;g__Streptococcus | 1 | 8 | 74 |
| d__Bacteria;p__Patescibacteria;c__Saccharimonadia;o__Saccharimonadales;f__Saccharimonadales;g__Saccharimonadales;s__uncultured_cyanobacterium | 1 | 8 | 74 |
| d__Bacteria;p__Proteobacteria;c__Gammaproteobacteria;o__Burkholderiales;f__Comamonadaceae;g__Tepidimonas | 1 | 8 | 61 |
| d__Bacteria;p__Proteobacteria;c__Gammaproteobacteria;o__Enterobacterales;f__Enterobacteriaceae;g__Escherichia-Shigella | 1 | 8 | 26 |
| d__Bacteria;p__Proteobacteria;c__Gammaproteobacteria;o__Vibrionales;f__Vibrionaceae;g__Vibrio | 1 | 8 | 13 |
