# Supplementary material for: Gallbladder microbiota in healthy dogs and dogs with mucocele formation
Source: PLoS One. 2023 Feb 10;18(2):e0281432. doi: 10.1371/journal.pone.0281432 (PMC9916591; doi:10.1371/journal.pone.0281432)
Supplement: S3 Table — Results of conventional cytology, aerobic and anaerobic bacterial culture, and eubacterial fluorescence in-situ hybridization (FISH) are also shown for each sample. (DOCX) [file pone.0281432.s003.docx]

| **Sample ID** | **Control 12** |  | **Control 15** |  | **Control 16** |  | **Control 18** |  |
| --- | --- | --- | --- | --- | --- | --- | --- | --- |
|  | 4 yr intact male Hound |  | 1 yr castrated male Staffordshire Terrier Mix | | 2 yr intact female Beagle |  | 3 yr intact male Staffordshire Terrier Mix | |
| **Cytology** | No bacteria |  | No bacteria |  | No bacteria |  | No bacteria |  |
| **Culture** | No growth |  | No growth |  | No growth |  | No growth |  |
| **F.I.S.H.** | Negative |  | Negative |  | Negative |  | Negative |  |
| **16S** | **ASV No Phenol (NP)** | **% Abund** | **ASV No Phenol (NP)** | **% Abund** | **ASV No Phenol (NP)** | **% Abund** | **ASV No Phenol (NP)** | **% Abund** |
|  | g_Meiothermus | 42.32 | g_Massilia | 63.05 | g_Geobacillus | 65.70 | g_Streptococcus | 73.62 |
|  | Clostridium sensu stricto 9 | 21.93 | g_Acidiphilium | 21.67 | Brevibacillus thermoruber | 15.53 | g_Escherichia-Shigella | 26.38 |
|  | g_Sphingobacterium | 15.35 | g_Stenotrophomonas | 15.27 | Lactobacillus | 5.79 |  |  |
|  | Carnobacterium | 9.65 |  |  | g_Chloroplast | 4.75 |  |  |
|  | f_Chitinophagaceae | 5.04 |  |  | g_Novosphingobium | 4.63 |  |  |
|  | g_Hymenobacter | 2.85 |  |  | g_Lysinibacillus | 2.56 |  |  |
|  | o_Bacillales | 2.41 |  |  | Eubacterium brachy | 1.04 |  |  |
|  | f_Spirochaetaceae | 0.44 |  |  |  |  |  |  |
|  | **TOTAL READS** | **456** | **TOTAL READS** | **203** | **TOTAL READS** | **6374** | **TOTAL READS** | **157730** |
|  |  |  |  |  |  |  |  |  |
|  | **ASV Phenol (P)** | **% Abund** | **ASV Phenol (P)** | **% Abund** | **ASV Phenol (P)** | **% Abund** | **ASV Phenol (P)** | **% Abund** |
|  | f_Enterobacteriaceae | 35.67 | f_Spirochaetaceae | 100 | Candidatus Finniella | 52.03 | f__Enterobacteriaceae | 59.62 |
|  | g_Gardnerella | 25.52 |  |  | d_Bacteria | 28.04 | g_Bacillus | 12.63 |
|  | g_[Ruminococcus] gauvreauii group | 23.19 |  |  | g_Psychromonas | 14.76 | Lactobacillus brevis | 4.90 |
|  | g_Vibrio | 13.09 |  |  | g_Novosphingobium | 5.17 | g_Curvibacter | 3.23 |
|  | f_Spirochaetaceae | 1.82 |  |  |  |  | g_Delftia | 3.02 |
|  | g_Chroococcidiopsis SAG 2023 | 0.45 |  |  |  |  | g_Listeria | 2.68 |
|  | g_Neisseria | 0.25 |  |  |  |  | g_Muribaculaceae | 2.54 |
|  |  |  |  |  |  |  | g_Enhydrobacter | 2.27 |
|  |  |  |  |  |  |  | f_Intrasporangiaceae | 2.18 |
|  | **TOTAL READS** | **1979** | **TOTAL READS** | **3** | **TOTAL READS** | **271** | g_Alishewanella | 1.90 |
|  |  |  |  |  |  |  | f_Dermatophilaceae | 1.47 |
|  |  |  |  |  |  |  | g_Novosphingobium | 1.37 |
|  |  |  |  |  |  |  | g_Pseudactinotalea | 0.80 |
|  |  |  |  |  |  |  | g_Turicella | 0.57 |
|  |  |  |  |  |  |  | Campylobacter ureolyticus | 0.38 |
|  |  |  |  |  |  |  | g_Qipengyuania | 0.29 |
|  |  |  |  |  |  |  | Capnocytophaga ochracea | 0.15 |
|  |  |  |  |  |  |  | **TOTAL READS** | **14294** |

| **Sample ID** | **Control 19** |  | **Control 21** |  | **Control 24** |  | **Control 25** |  |
| --- | --- | --- | --- | --- | --- | --- | --- | --- |
|  | **2 yr castrated male Staffordshire Terrier Mix** | | **3 yr intact male German Shepherd** | | **3 yr intact male Border Collie Mix** | | **4 yr intact male Hound** |  |
| **Cytology** | Yeast |  | No bacteria |  | No bacteria |  | No bacteria |  |
| **Culture** | No growth |  | No growth |  | No growth |  | No growth |  |
| **F.I.S.H.** | Negative |  | Negative |  | Negative |  | Negative |  |
| **16S** | **ASV No Phenol (NP)** | **% Abundance** | **ASV No Phenol (NP)** | **% Abundance** | **ASV No Phenol (NP)** | **% Abundance** | **ASV No Phenol (NP)** | **% Abundance** |
|  | g_Pseudomonas | 65.90 | Unassigned | 64.30 | g_Geobacillus | 92.11 | g_Bacillus | 38.45 |
|  | Candidatus Obscuribacter | 12.91 | d_Bacteria | 12.33 | g_Chloroplast | 2.26 | g_Paracoccus | 27.50 |
|  | g_Limnochordaceae | 6.57 | g_Novosphingobium | 8.82 | f_Peptostreptococcaceae | 1.59 | Lactococcus lactis | 25.69 |
|  | Flavobacterium Cytophaga sp. | 5.37 | g_Bacteroides | 8.05 | g_Candidatus Finniella | 1.49 | Acinetobacter radioresistens | 5.33 |
|  | Botryosphaeria dothidea | 4.96 | g_WD2101 soil group | 6.51 | g_Haliangium | 1.44 | g_Acinetobacter uncultured rumen | 3.03 |
|  | g_Achromobacter | 2.31 |  |  | g_Stenotrophomonas | 0.84 |  |  |
|  | f_Isosphaeraceae | 1.79 |  |  | g_Massilia | 0.27 |  |  |
|  | g_Bacteroides | 0.19 |  |  |  |  |  |  |
|  | **TOTAL READS** | **3686** | **TOTAL READS** | **1168** | **TOTAL READS** | **12983** | **TOTAL READS** | **2476** |
|  |  |  |  |  |  |  |  |  |
|  | **ASV Phenol (P)** | **% Abundance** | **ASV Phenol (P)** | **% Abundance** | **ASV Phenol (P)** | **% Abundance** | **ASV Phenol (P)** | **% Abundance** |
|  | g_Granulicatella | 67.00 | Unassigned | 77.31 | g_Saccharimonadales uncultured cyanobacterium | 74.44 | g_Pseudomonas | 67.00 |
|  | g_Rhodococcus | 33.00 | f_Enterobacteriaceae | 14.35 | g_Mitochondria | 25.56 | g_Cloacibacterium | 11.50 |
|  |  |  | d_Bacteria | 5.96 |  |  | g_Delftia | 9.56 |
|  |  |  | d_Eukaryota | 1.22 |  |  | g_Serratia | 6.86 |
|  |  |  | g_Aeromonas | 1.16 |  |  | g_Turicella | 1.99 |
|  |  |  |  |  |  |  | g_Curvibacter | 1.06 |
|  |  |  |  |  |  |  | g_Romboutsia | 0.91 |
|  |  |  |  |  |  |  | Bacillus halodurans | 0.83 |
|  |  |  |  |  |  |  | Enterococcus cecorum | 0.28 |
|  | **TOTAL READS** | **300** | **TOTAL READS** | **3610** | **TOTAL READS** | **356** | **TOTAL READS** | **3861** |

| **Sample ID** | **Control 3** |  | **Control 30** |  | **Control 36** |  | **Control 5** |  | **Control 7** |  |
| --- | --- | --- | --- | --- | --- | --- | --- | --- | --- | --- |
|  | **4 yr intact male Hound** | | **2.5 yr intact male Staffordshire Terrier Mix** | | **1.5 yr intact male Staffordshire Terrier Mix** | | **2 yr intact male Staffordshire Terrier Mix** | | **2 yr intact female Staffordshire Terrier Mix** | |
| **Cytology** | No bacteria |  | No bacteria |  | No bacteria |  | No bacteria |  | Yeast |  |
| **Culture** | No growth |  | No growth |  | No growth |  | No growth |  | No growth |  |
| **F.I.S.H.** | Negative |  | Negative |  | Negative |  | Negative |  | Negative |  |
| **16S** | **ASV No Phenol (NP)** | **% Abundance** | **ASV No Phenol (NP)** | **% Abundance** | **ASV No Phenol (NP)** | **% Abundance** | **ASV No Phenol (NP)** | **% Abundance** | **ASV No Phenol (NP)** | **% Abundance** |
|  | g_Bacillus | 29.01 | g_Geobacillus | 81.88 | g_Veillonella | 34.12 | g_Geobacillus | 53.27 | Unassigned | 86.52 |
|  | Unassigned | 21.88 | g_Stenotrophomonas | 10.75 | g_Peptoniphilus | 33.48 | Brevibacillus thermoruber | 45.78 | d_Bacteria | 5.65 |
|  | g_Acidaminococcus | 15.24 | g_Curvibacter | 7.37 | Bacillus halodurans | 18.55 | g_Ralstonia | 0.27 | g_Chloroplast | 4.63 |
|  | o_Micavibrionales | 11.10 |  |  | g_Chryseobacterium | 13.86 | g_Achromobacter | 0.25 | Corynebacterium kroppenstedtii | 1.53 |
|  | g_Gardnerella | 10.26 |  |  |  |  | g_Fenollaria | 0.25 | g_Massilia | 1.37 |
|  | g_Curvibacter | 8.87 |  |  |  |  | g_Enhydrobacter | 0.10 | d_Eukaryota | 0.29 |
|  | Bacillus halodurans | 2.90 |  |  |  |  | g_Veillonella | 0.075 |  |  |
|  | g_Aeromonas | 0.69 |  |  |  |  | g_Peptoniphilus | 0.006 |  |  |
|  | g_Muribaculaceae | 0.042 |  |  |  |  |  |  |  |  |
|  | **TOTAL READS** | **4757** | **TOTAL READS** | **2252** | **TOTAL READS** | **469** | **TOTAL READS** | **34566** | **TOTAL READS** | **5096** |
|  |  |  |  |  |  |  |  |  |  |  |
|  | **ASV Phenol (P)** | **% Abundance** | **ASV Phenol (P)** | **% Abundance** | **ASV Phenol (P)** | **% Abundance** | **ASV Phenol (P)** | **% Abundance** | **ASV Phenol (P)** | **% Abundance** |
|  | Unassigned | 83.27 | g_Neisseria | 27.41 | g_Nocardioides | 36.30 | g_Tepidimonas | 60.81 | Unassigned | 86.69 |
|  | g_Bacillus | 11.20 | g_Oryzihumus | 26.21 | Bacillus coagulans | 25.12 | g_Flectobacillus | 36.28 | d_Bacteria | 10.11 |
|  | d_Bacteria | 3.25 | Prevotella melaninogenica | 21.05 | g_Anaerobacillus | 14.30 | g_Stenotrophomonas | 2.92 | g_Ruminiclostridium | 1.76 |
|  | d_Eukaryota | 1.15 | g_Micrococcus | 11.62 | g_SM1A02 | 9.86 |  |  | g_Solirubrobacter | 1.44 |
|  | Bacillus halodurans | 1.13 | g_Haemophilus | 8.33 | g_Hyphomicrobium | 9.38 |  |  |  |  |
|  |  |  | Pandoraea Alcaligenes sp. | 3.73 | g_Veillonella | 2.76 |  |  |  |  |
|  |  |  | g_Sphingobacterium | 1.64 | g_Sporichthya | 2.28 |  |  |  |  |
|  | **TOTAL READS** | **5749** | **TOTAL READS** | **912** | **TOTAL READS** | **832** | **TOTAL READS** | **1439** | **TOTAL READS** | **6528** |
