# Supplementary material for: Gallbladder microbiota in healthy dogs and dogs with mucocele formation
Source: PLoS One. 2023 Feb 10;18(2):e0281432. doi: 10.1371/journal.pone.0281432 (PMC9916591; doi:10.1371/journal.pone.0281432)
Supplement: S4 Table — (DOCX) [file pone.0281432.s004.docx]

**S4 Table** Thirty one amplicon sequence variants amplified from gallbladder mucus collected from 13 dogs diagnosed with mucocele formation that were also amplified from ≥ 1 negative extraction control sample but at lower abundance than observed in mucus.

| **Amplicon Sequence Variants** | **Dogs (n=13)** | | **Max % Abundance** |
| --- | --- | --- | --- |
|  | **No.** | **%** |  |
| d__Bacteria | 8 | 61.5 | 16.63 |
| Unassigned | 8 | 61.5 | 96.35 |
| d__Eukaryota | 6 | 46.2 | 1.14 |
| d__Bacteria;p__Cyanobacteria;c__Cyanobacteriia;o__Chloroplast;f__Chloroplast;g__Chloroplast | 4 | 30.8 | 5.99 |
| d__Bacteria;p__Firmicutes;c__Bacilli;o__Bacillales;f__Bacillaceae;g__Bacillus | 4 | 30.8 | 29.27 |
| d__Bacteria;p__Firmicutes;c__Bacilli;o__Bacillales;f__Bacillaceae;g__Geobacillus | 4 | 30.8 | 66.40 |
| d__Bacteria;p__Proteobacteria;c__Gammaproteobacteria;o__Enterobacterales;f__Enterobacteriaceae;g__Escherichia-Shigella | 3 | 23.1 | 99.98 |
| d__Bacteria;p__Firmicutes;c__Bacilli;o__Lactobacillales;f__Streptococcaceae;g__Streptococcus | 2 | 15.4 | 77.60 |
| d__Bacteria;p__Actinobacteriota;c__Actinobacteria;o__Micrococcales;f__Micrococcaceae;g__Micrococcus | 2 | 15.4 | 15.01 |
| d__Bacteria;p__Deinococcota;c__Deinococci;o__Thermales;f__Thermaceae;g__Thermus | 2 | 15.4 | 5.61 |
| d__Bacteria;p__Firmicutes;c__Bacilli;o__Brevibacillales;f__Brevibacillaceae;g__Brevibacillus;s__Brevibacillus_thermoruber | 2 | 15.4 | 30.48 |
| d__Bacteria;p__Firmicutes;c__Clostridia;o__Clostridiales;f__Clostridiaceae;g__Clostridium_sensu_stricto_1;s__Clostridium_perfringens | 2 | 15.4 | 52.41 |
| d__Bacteria;p__Proteobacteria;c__Gammaproteobacteria;o__Enterobacterales;f__Enterobacteriaceae | 2 | 15.4 | 86.87 |
| d__Bacteria;p__Proteobacteria;c__Gammaproteobacteria;o__Pseudomonadales;f__Pseudomonadaceae;g__Pseudomonas | 1 | 7.7 | 10.82 |
| d__Bacteria;p__Actinobacteriota;c__Actinobacteria;o__Bifidobacteriales;f__Bifidobacteriaceae;g__Bifidobacterium | 1 | 7.7 | 0.56 |
| d__Bacteria;p__Bacteroidota;c__Bacteroidia;o__Flavobacteriales;f__Weeksellaceae | 1 | 7.7 | 4.73 |
| d__Bacteria;p__Firmicutes;c__Bacilli;o__Lactobacillales;f__Enterococcaceae;g__Enterococcus | 1 | 7.7 | 100.00 |
| d__Bacteria;p__Firmicutes;c__Bacilli;o__Lactobacillales;f__Lactobacillaceae;g__Lactobacillus | 1 | 7.7 | 6.13 |
| d__Bacteria;p__Firmicutes;c__Bacilli;o__Lactobacillales;f__Lactobacillaceae;g__Lactobacillus;s__Lactobacillus_fermentum | 1 | 7.7 | 8.72 |
| d__Bacteria;p__Firmicutes;c__Bacilli;o__Lactobacillales;f__Lactobacillaceae;g__Lactobacillus;s__Lactobacillus_rhamnosus | 1 | 7.7 | 4.14 |
| d__Bacteria;p__Firmicutes;c__Bacilli;o__Paenibacillales;f__Paenibacillaceae;g__Paenibacillus;s__Paenibacillus_alginolyticus | 1 | 7.7 | 2.30 |
| d__Bacteria;p__Patescibacteria;c__Saccharimonadia;o__Saccharimonadales;f__Saccharimonadales;g__Saccharimonadales;s__uncultured_cyanobacterium | 1 | 7.7 | 11.51 |
| d__Bacteria;p__Proteobacteria;c__Alphaproteobacteria;o__Paracaedibacterales;f__Paracaedibacteraceae;g__Candidatus_Finniella;s__uncultured_bacterium | 1 | 7.7 | 0.37 |
| d__Bacteria;p__Proteobacteria;c__Alphaproteobacteria;o__Sphingomonadales;f__Sphingomonadaceae;g__Sphingobium | 1 | 7.7 | 46.79 |
| d__Bacteria;p__Proteobacteria;c__Alphaproteobacteria;o__Sphingomonadales;f__Sphingomonadaceae;g__Sphingomonas | 1 | 7.7 | 2.59 |
| d__Bacteria;p__Proteobacteria;c__Gammaproteobacteria;o__Burkholderiales;f__Comamonadaceae | 1 | 7.7 | 15.23 |
| d__Bacteria;p__Proteobacteria;c__Gammaproteobacteria;o__Burkholderiales;f__Comamonadaceae;g__Curvibacter | 1 | 7.7 | 5.55 |
| d__Bacteria;p__Proteobacteria;c__Gammaproteobacteria;o__Burkholderiales;f__Comamonadaceae;g__Variovorax | 1 | 7.7 | 5.39 |
| d__Bacteria;p__Proteobacteria;c__Gammaproteobacteria;o__Enterobacterales;f__Morganellaceae;g__Morganella | 1 | 7.7 | 1.44 |
| d__Bacteria;p__Proteobacteria;c__Gammaproteobacteria;o__Enterobacterales;f__Yersiniaceae | 1 | 7.7 | 7.09 |
| d__Bacteria;p__Proteobacteria;c__Gammaproteobacteria;o__Pseudomonadales;f__Moraxellaceae;g__Acinetobacter | 1 | 7.7 | 13.19 |
