# Supplementary material for: Gallbladder microbiota in healthy dogs and dogs with mucocele formation
Source: PLoS One. 2023 Feb 10;18(2):e0281432. doi: 10.1371/journal.pone.0281432 (PMC9916591; doi:10.1371/journal.pone.0281432)
Supplement: S5 Table — Results of aerobic and anaerobic bacterial culture, and eubacterial fluorescence in-situ hybridization (FISH) are also shown for each sample. (DOCX) [file pone.0281432.s005.docx]

| **Sample ID** | **Mucocele 13** |  | **Mucocele 22** |  | **Mucocele 26** |  | **Mucocele 2** |  |
| --- | --- | --- | --- | --- | --- | --- | --- | --- |
|  | **13 yr castrated male Shetland sheepdog** | | **12 yr spayed female American Cocker Spaniel** | | **11 yr castrated male Labrador Retriever** | | **13 yr castrated male Shetland Sheepdog** | |
| **Culture** | *Escherichia coli* |  | *Escherichia coli* |  | *Escherichia coli* |  | *Enterococcus* |  |
| **F.I.S.H.** | Not performed |  | Negative |  | Positive (Gram negative) |  | Positive (Gram positive) | |
| **Antibiotic history** | Undetermined |  | Positive |  | Negative |  | Negative |  |
| **16S** | **ASV No Phenol (NP)** | **% Abundance** | **ASV No Phenol (NP)** | **% Abundance** | **ASV No Phenol (NP)** | **% Abundance** | **ASV No Phenol (NP)** | **% Abundance** |
|  | g_Escherichia-Shigella | 99.98 | Unassigned | 84.82 | g_Streptococcus | 73.02 | g_Ochrobactrum | 60 |
|  | Bacteroides vulgatus | 0.015 | d_Bacteria | 6.13 | g_Escherichia-Shigella | 26.96 | g_Muribaculaceae | 40 |
|  | Campylobacter ureolyticus | 0.0035 | g_Chloroplast | 5.99 | Veillonella montpellierensis | 0.019 |  |  |
|  |  |  | g_Bacteroides | 1.04 | Lactobacillus brevis | 0.0069 |  |  |
|  |  |  | Lactobacillus brevis | 0.85 |  |  |  |  |
|  |  |  | d_Eukaryota | 0.57 |  |  |  |  |
|  |  |  | Parabacteroides merdae | 0.24 |  |  |  |  |
|  |  |  | g_Mitochondria Rhynchosporium secalis | 0.22 |  |  |  |  |
|  |  |  | Parabacteroides distasonis | 0.14 |  |  |  |  |
|  | **TOTAL READS** | **115481** | **TOTAL READS** | **5073** | **TOTAL READS** | **144164** | **TOTAL READS** | **5** |
|  |  |  |  |  |  |  |  |  |
|  | **ASV Phenol (P)** | **% Abundance** | **ASV Phenol (P)** | **% Abundance** | **ASV Phenol (P)** | **% Abundance** | **ASV Phenol (P)** | **% Abundance** |
|  | g_Escherichia-Shigella | 99.60 | f_Enterobacteriaceae | 86.87 | g_Streptococcus | 68.73 | g_Enterococcus | 100 |
|  | Unassigned | 0.36 | Unassigned | 12.07 | g_Escherichia-Shigella | 31.27 |  |  |
|  | Bacteroides vulgatus | 0.032 | g_Enhydrobacter | 0.40 |  |  |  |  |
|  | d_Bacteria | 0.0092 | g_Eggerthella | 0.33 |  |  |  |  |
|  | Corynebacterium aurimucosum | 0.00052 | d_Eukaryota | 0.20 |  |  |  |  |
|  | o_Enterobacterales | 0.00048 | f_Verrucomicrobiaceae | 0.12 |  |  |  |  |
|  | d_Eukaryota | 0.00011 |  |  |  |  |  |  |
|  | g_Ralstonia | 0.00011 |  |  |  |  |  |  |
|  | g_Haemophilus | 0.000068 |  |  |  |  |  |  |
|  | **TOTAL READS** | **17585627** | **TOTAL READS** | **8921** | **TOTAL READS** | **17343** | **TOTAL READS** | **385312** |

| **Sample ID** | **Mucocele 35** |  | **Mucocele 20** |  | **Mucocele 10** |  | **Mucoccele 9** |  |
| --- | --- | --- | --- | --- | --- | --- | --- | --- |
|  | **11 yr castrated male Chihuahua** | | **12 yr spayed female Hound** | | **11 yr spayed female Chihuahua** | | **11 yr castrated male Mixed Breed** | |
| **Culture** | Not performed |  | No growth |  | No growth |  | No growth |  |
| **F.I.S.H.** | DAPI Positive/Eub Negative (Gram positive) | | Negative |  | Negative |  | Negative |  |
| **Antibiotic history** | Positive |  | Negative |  | Undetermined |  | Negative |  |
| **16S** | **ASV No Phenol (NP)** | **% Abundance** | **ASV No Phenol (NP)** | **% Abundance** | **ASV No Phenol (NP)** | **% Abundance** | **ASV No Phenol (NP)** | **% Abundance** |
|  | Clostridium perfringens | 52.41 | g_Geobacillus | 22.46 | g_Geobacillus | 66.40 | g_Bacillus | 29.27 |
|  | g_Escherichia-Shigella | 41.76 | g_Chroococcidiopsis SAG 2023 uncultured cyanobacterium | 21.01 | Brevibacillus thermoruber | 30.48 | g_Micrococcus | 15.01 |
|  | Parabacteroides distasonis | 5.83 | g_Acinetobacter | 13.19 | g_Chloroplast | 2.45 | Veillonella montpellierensis | 8.55 |
|  |  |  | g_Bacillus | 11.80 | g_Mitochondria | 0.27 | d_Bacteria | 6.06 |
|  |  |  | g_Pseudomonas | 10.82 | g_Flavobacterium | 0.21 | g_Enhydrobacter | 5.11 |
|  |  |  | f_Yersiniaceae | 7.09 | Rhodococcus aerolatus | 0.14 | g_Dietzia | 4.91 |
|  |  |  | g_Chloroplast | 5.32 | g_Novosphingobium | 0.044 | g_Propioniciclava | 3.36 |
|  |  |  | Sphingomonas koreensis | 4.18 |  |  | Stenotrophomonas rhizophila | 3.30 |
|  |  |  | g_Thermus | 2.40 |  |  | g_Cellvibrio | 2.96 |
|  |  |  | g_Kocuria | 1.37 |  |  | g_Peptoniphilus | 2.36 |
|  |  |  | f_Chroococcidiopsaceae | 0.35 |  |  | f_Neisseriaceae | 2.22 |
|  |  |  | g_Achromobacter | 0.0063 |  |  | g_Chryseobacterium | 2.09 |
|  | **TOTAL READS** | **15564** | **TOTAL READS** | **79127** | **TOTAL READS** | **11447** | g_BIrii41 | 2.09 |
|  |  |  |  |  |  |  | g_Mitochondria Sclerotinia borealis | 2.02 |
|  | **ASV Phenol (P)** | **% Abundance** | **ASV Phenol (P)** | **% Abundance** | **ASV Phenol (P)** | **% Abundance** | g_Salinicoccus | 1.95 |
|  |  |  | g_Sphingobium | 46.79 | Lactobacillus brevis | 52.45 | g_Saccharimonadaceae | 1.62 |
|  |  |  | f_Enterobacteriaceae | 16.86 | g_Peptoniphilus | 8.08 | g_Mitochondria | 1.48 |
|  |  |  | f_Comamonadaceae | 15.23 | Bacillus halodurans | 7.33 | g_Salinicoccus | 1.35 |
|  |  |  | g_Variovorax | 5.39 | g_Micrococcus | 6.49 | g_Chloroplast uncultured Streptophyta | 1.14 |
|  |  |  | g_Pseudomonas | 3.25 | g_Lactobacillus | 6.13 | Anaerococcus hydrogenalis | 1.14 |
|  |  |  | g_Sphingomonas | 2.59 | f__Lachnospiraceae | 4.27 | g_Aerococcus | 0.81 |
|  |  |  | Paenibacillus alginolyticus | 2.30 | Lactobacillus rhamnosus | 4.14 | g_Leucobacter | 0.74 |
|  |  |  | g_Morganella | 1.44 | g_Ochrobactrum | 3.23 | g_Delftia | 0.47 |
|  |  |  | g_Turicella | 1.36 | g_Vagococcus | 3.14 | **TOTAL READS** | **1486** |
|  |  |  | g_Chloroplast | 1.33 | g_ADurb.Bin063-1 | 2.45 |  |  |
|  |  |  | g_JG36-TzT-191 | 0.78 | Bacillus alcalophilus | 0.60 | **ASV Phenol (P)** | **% Abundance** |
|  |  |  | g_Friedmanniella | 0.66 | g_Bacteroides | 0.49 | Lactobacillus brevis | 21.72 |
|  |  |  | g_Bifidobacterium | 0.56 | f_Dermacoccaceae | 0.39 | Unassigned | 14.36 |
|  |  |  | g_Novosphingobium | 0.54 | g_Fenollaria | 0.36 | g_Saccharimonadales uncultured cyanobacterium | 11.51 |
|  |  |  | g_Candidatus Finniella | 0.37 | Corynebacterium kroppenstedtii | 0.26 | Lactobacillus fermentum | 8.72 |
|  |  |  | Clostridium perfringens | 0.22 | g_Erysipelatoclostridium | 0.13 | g_Paracoccus | 8.41 |
|  |  |  | g_Flaviaesturariibacter | 0.21 | g_Chryseobacterium | 0.048 | g_Thermus | 5.61 |
|  |  |  | Bacillus alcalophilus | 0.071 |  |  | g_Curvibacter | 5.55 |
|  |  |  | Sphingomonas koreensis | 0.054 |  |  | f_Weeksellaceae | 4.73 |
|  | **TOTAL READS** | **0** | **TOTAL READS** | **76155** | **TOTAL READS** | **8379** | Blastococcus aggregatus | 3.80 |
|  |  |  |  |  |  |  | d_Bacteria | 2.99 |
|  |  |  |  |  |  |  | Enterococcus cecorum | 2.86 |
|  |  |  |  |  |  |  | f_Nocardioidaceae | 2.84 |
|  |  |  |  |  |  |  | g_Desulfosporosinus | 1.93 |
|  |  |  |  |  |  |  | g_Pseudonocardia | 1.31 |
|  |  |  |  |  |  |  | g_Steroidobacter | 1.29 |
|  |  |  |  |  |  |  | g_Ralstonia | 0.91 |
|  |  |  |  |  |  |  | Veillonella montpellierensis | 0.89 |
|  |  |  |  |  |  |  | g_Blastococcus | 0.56 |
|  |  |  |  |  |  |  | **TOTAL READS** | **5493** |

| **Sample ID** | **Mucocele 27** |  | **Mucocele 1** |  | **Mucocele 33** |  | **Mucocele 4** |  | **Mucocele 8** |  |
| --- | --- | --- | --- | --- | --- | --- | --- | --- | --- | --- |
|  | **10 yr castrated male Bichon Frise** | | **13 yr castrated male Yorkshire Terrier** | | **13 yr castrated male Chihuahua** | | **5 yr spayed female Maltese** | | **16 yr castrated male Jack Russell Terrier** | |
| **Culture** | No growth |  | No growth |  | No growth |  | No growth |  | No growth |  |
| **F.I.S.H.** | DAPI Positive/Eub Negative (Gram not performed) | | Negative |  | Negative |  | Negative |  | Negative |  |
| **Antibiotic history** | Undetermined |  | Positive |  | Positive |  | Positive |  | Undetermined |  |
| **16S** | **ASV No Phenol (NP)** | **% Abundance** | **ASV No Phenol (NP)** | **% Abundance** | **ASV No Phenol (NP)** | **% Abundance** | **ASV No Phenol (NP)** | **% Abundance** | **ASV No Phenol (NP)** | **% Abundance** |
|  | Unassigned | 96.35 | Unassigned | 73.20 | Unassigned | 85.70 | g_Geobacillus | 53.24 |  |  |
|  | d_Bacteria | 3.38 | g_Geobacillus | 20.79 | d_Bacteria | 13.31 | g_Bacillus | 23.50 |  |  |
|  | d_Eukaryota | 0.27 | d_Bacteria | 3.06 | d_Eukaryota | 0.99 | Brevibacillus thermoruber | 4.35 |  |  |
|  |  |  | g_Aggregatibacter | 0.73 |  |  | g_Chloroplast | 4.13 |  |  |
|  |  |  | Stenotrophomonas nitritireducens | 0.54 |  |  | g_Abiotrophia | 2.73 |  |  |
|  |  |  | g_Dermabacter | 0.41 |  |  | g_Mitochondria | 2.31 |  |  |
|  |  |  | f_Pirellulaceae | 0.41 |  |  | g_Ralstonia | 1.99 |  |  |
|  |  |  | d_Eukaryota | 0.37 |  |  | Acinetobacter radioresistens | 1.89 |  |  |
|  |  |  | g_Mitochondria | 0.29 |  |  | d_Bacteria | 1.86 |  |  |
|  |  |  | o_Micrococcales | 0.20 |  |  | g_Peptoniphilus | 1.52 |  |  |
|  |  |  |  |  |  |  | g_Polaromonas | 1.19 |  |  |
|  |  |  |  |  |  |  | g_Reyranella | 0.94 |  |  |
|  |  |  |  |  |  |  | g_Mitochondria Clonostachys rosea | 0.35 |  |  |
|  | **TOTAL READS** | **5148** | **TOTAL READS** | **5915** | **TOTAL READS** | **1420** | **TOTAL READS** | **5875** | **TOTAL READS** | **0** |
|  |  |  |  |  |  |  |  |  |  |  |
|  | **ASV Phenol (P)** | **% Abundance** | **ASV Phenol (P)** | **% Abundance** | **ASV Phenol (P)** | **% Abundance** | **ASV Phenol (P)** | **% Abundance** | **ASV Phenol (P)** | **% Abundance** |
|  | g_Streptococcus | 77.60 | Unassigned | 81.69 | Unassigned | 86.05 | Unassigned | 94.76 | Unassigned | 91.89 |
|  | Unassigned | 21.46 | d_Bacteria | 16.63 | d_Bacteria | 12.80 | d_Bacteria | 4.67 | d_Bacteria | 3.62 |
|  | d_Bacteria | 0.84 | f_Alcaligenaceae | 1.10 | d_Eukaryota | 1.14 | Dermacoccus nishinomiyaensis | 0.49 | Veillonella montpellierensis | 1.77 |
|  | d_Eukaryota | 0.10 | d_Eukaryota | 0.40 |  |  | g_Massilia | 0.079 | g_Bacillus | 1.75 |
|  |  |  | g_Peptoniphilus | 0.18 |  |  |  |  | d_Eukaryota | 0.51 |
|  |  |  |  |  |  |  |  |  | g_Undibacterium | 0.45 |
|  | **TOTAL READS** | **41149** | **TOTAL READS** | **3271** | **TOTAL READS** | **2796** | **TOTAL READS** | **6357** | **TOTAL READS** | **25942** |
